# When more is not more: evidence for bounded perceptual-relational integration in non-symbolic proportion judgments

**DOI:** 10.3389/fpsyg.2026.1904042

**Published:** 2026-09-30

**Authors:** Qiang Wu, Di Zhang, Lei Wu, Liang Zhi Jia, Yun Pan

**Affiliations:** 1 School of Psychology, Guizhou Normal University, Guiyang, China; 2 School of Literature and Communication, Qiannan Normal University for Nationalities, Duyun, China

**Keywords:** bounded integration, drift-diffusion models, non-symbolic proportion judgments, numerical cognition, perceptual-relational integration

## Abstract

Non-symbolic proportion judgments require relational information to be extracted from perceptual input, yet the computational principles governing the contribution of continuous visual magnitude remain unclear. The present study examined whether perceptual influences on proportion judgments vary with relational context and whether their contribution increases linearly with cue strength or is subject to functional constraints. Across four experiments, participants compared non-symbolic proportions while continuous perceptual magnitude was manipulated through area scaling without altering the underlying proportional relationships. Experiments 1 and 2 showed that unilateral enlargement and contraction both influenced proportion judgments, indicating that perceptual effects were not specific to a single direction of physical scaling. Experiment 3 further showed that performance varied according to the alignment between perceptual and relational information, with perceptually congruent conditions generally facilitating performance and incongruent conditions producing interference. Experiment 4 manipulated perceptual cue strength at two scaling levels and found that increasing cue magnitude did not produce proportional increases in behavioral influence; this constraint was more evident in response-time dynamics than in categorical accuracy. Hierarchical drift-diffusion modeling provided converging computational evidence: a model incorporating a bounded transformation of perceptual input showed better predictive performance than a purely linear integration model. Together, these findings indicate that non-symbolic proportion judgments are not fully insulated from continuous perceptual variation. Instead, perceptual information contributes to decision formation in a context-sensitive manner, and its influence is better characterized by a constrained computational framework than by unrestricted linear cue accumulation.

## Introduction

Human cognition routinely requires extracting abstract relational structure from perceptual input. Whether estimating the probability of rainfall from a weather graphic, evaluating risk from epidemiological data, or comparing proportions in visual displays, observers must extract relational structure while simultaneously processing continuous visual magnitude cues such as size, area, and spatial extent (Gigerenzer and Edwards, 2003; Peters et al., 2006). A central question is therefore whether non-symbolic proportion judgments rely on abstract relational representations that are insulated from perceptual variation, or whether they emerge from dynamic integration processes that remain sensitive to continuous visual information (Walsh, 2003).

Non-symbolic proportion judgments provide an ideal paradigm for investigating this issue because they require observers to compare relational quantities without relying on symbolic notation. In a typical task, participants determine which of two visual displays contains a larger proportion of a target component while the underlying relational structure is conveyed entirely through perceptual information. Sensitivity to proportional relationships emerges early in development and has been observed across different species, suggesting that proportion processing reflects a fundamental quantitative ability (Boyer et al., 2008; Cantlon et al., 2006; McCrink and Wynn, 2007; Vallentin and Nieder, 2008). At the same time, proportion judgments are theoretically distinct from absolute quantity estimation because they require encoding relations between magnitudes rather than individual magnitudes themselves (Siegler and Lortie-Forgues, 2015).

One influential account proposes that proportions are represented through an abstract relational system that is at least partially separable from the physical properties of constituent quantities. The Ratio Processing System (RPS) framework suggests that ratios are encoded as relational magnitudes that remain relatively invariant across transformations preserving the underlying proportional structure (Matthews and Chesney, 2015; Matthews et al., 2016; Park et al., 2021). Consistent with this view, successful comparisons between non-symbolic proportions and symbolic fractions have been interpreted as evidence that proportion representations can be accessed independently of specific perceptual features (Matthews and Chesney, 2015; McCrink and Wynn, 2007; Siegler et al., 2011).

However, accumulating evidence from numerical cognition challenges the assumption that quantitative judgments are fully insulated from perceptual variation. Research on non-symbolic quantity processing has shown that continuous perceptual properties, including cumulative area, density, and spatial extent, can systematically influence judgments even when these properties are irrelevant to the task requirements (Aulet and Lourenco, 2023b; Boyer and Levine, 2012, 2015; Gebuis and Reynvoet, 2012a; Leibovich et al., 2017). Performance is often enhanced when perceptual cues are consistent with the relevant quantitative structure and impaired when they conflict with it (Boyer and Levine, 2015; Gevers et al., 2016; Gilmore et al., 2016). These findings suggest that quantitative judgments may involve interactions between relational representations and continuous perceptual information rather than relying exclusively on abstract representations.

Several theoretical perspectives have attempted to explain how perceptual information contributes to quantitative judgments. Magnitude-based accounts propose that numerical decisions may rely on noisy representations of quantitative information, whereas perceptual integration frameworks emphasize that judgments emerge from the combination of multiple continuous visual signals, including area, density, and spatial extent (Boyer and Levine, 2015; Gebuis and Reynvoet, 2012a; Leibovich et al., 2017). From this perspective, relational judgments are not necessarily independent of perceptual input; rather, perceptual information may provide additional evidence that is incorporated during decision formation.

Importantly, these perspectives do not require a strict dichotomy between abstract relational representations and perceptual influences. Instead, relational representations may define the target structure of the task, while continuous perceptual signals contribute supplementary evidence that is weighted during decision making. Thus, the critical question is not whether proportion judgments are exclusively ratio-based or magnitude-based, but rather how perceptual information is integrated with relational structure and what computational principles constrain this integration process.

A powerful approach for addressing this question is the ratio congruity paradigm, which examines whether task-irrelevant perceptual information systematically biases relational judgments. Previous studies have shown that proportion judgments are facilitated when perceptual cues are consistent with proportional relationships and disrupted when these cues conflict with the underlying relational structure (Matthews and Lewis, 2017). However, existing research has primarily focused on demonstrating the presence of perceptual bias, while the computational principles governing how perceptual influence changes with cue strength remain unclear.

A remaining theoretical challenge concerns the functional relationship between perceptual cue strength and behavioral influence. Although previous studies have established that irrelevant perceptual information can bias proportion judgments, less is known about how the magnitude of this influence changes as perceptual cues become stronger. This question is critical because perceptual integration mechanisms may not simply determine whether a cue contributes to decision making, but also regulate how strongly that cue affects the final judgment.

Under a standard linear integration account, perceptual influence should increase systematically with cue strength. When a perceptual signal becomes stronger or more reliable, it should receive greater weight during evidence accumulation, resulting in progressively larger facilitation or interference effects (Ernst and Banks, 2002; Ernst and Bülthoff, 2004). From this perspective, increasing the magnitude of perceptual differences should produce a monotonic increase in behavioral bias.

However, perceptual systems may not always benefit from unrestricted cue amplification. Alternative accounts based on normalization and ecologically constrained inference suggest that perceptual processing mechanisms regulate information integration according to contextual reliability and computational constraints (Carandini and Heeger, 2011; Knill and Pouget, 2004; Körding et al., 2007; Shams and Beierholm, 2010; Summerfield and Tsetsos, 2015). Under such frameworks, extremely strong or implausible perceptual signals may be down-weighted rather than continuously increasing their contribution to behavior. Applied to proportion cognition, these accounts predict a bounded or non-linear relationship between perceptual cue strength and behavioral influence, whereby moderate perceptual signals may exert stronger effects than excessively large or implausible cues.

To date, the possibility that perceptual influence in proportion judgments may be computationally constrained has not been directly examined. Existing research has primarily demonstrated that perceptual cues can bias quantitative judgments, but has rarely investigated whether the relationship between cue strength and behavioral influence follows a linear function or exhibits bounded dynamics. Consequently, the computational principles governing the integration of relational and perceptual information during proportion judgments remain unclear.

The present study addresses this issue by systematically manipulating perceptual cue strength while preserving the underlying proportional structure. A linear integration account predicts that increasing perceptual magnitude should continuously amplify congruity effects because stronger cues exert greater influence on decision formation. In contrast, a bounded integration account predicts that perceptual influence should eventually plateau or attenuate when cue magnitude becomes extreme, particularly when relational information provides sufficiently strong evidence for the correct judgment.

## Overview of the research

The present study addresses this issue by systematically manipulating the direction, symmetry, congruity, and magnitude of continuous perceptual signals within a unified non-symbolic proportion comparison paradigm. Across four experiments, we examine three key aspects of perceptual-relational integration (Figure 1).

**FIGURE 1:**
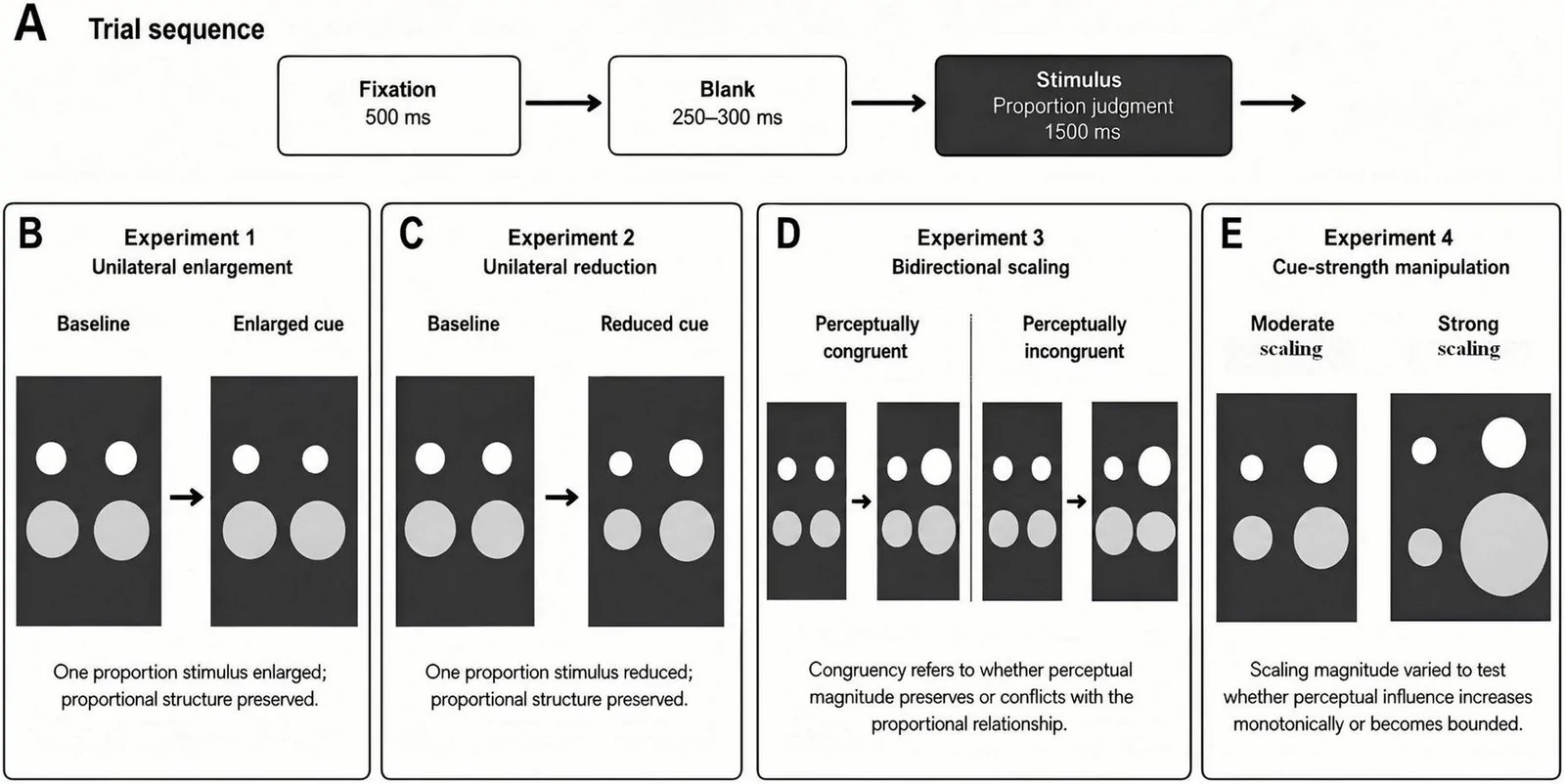
Experimental paradigm and perceptual magnitude manipulations across Experiments 1–4. **(A)**Trial sequence. Each trial began with a fixation display (500 ms), followed by a blank interval (250–300 ms) and a stimulus display (1500 ms). Participants judged which side contained the larger proportional relationship between the two components. **(B–E)** Schematic illustrations of perceptual magnitude manipulations across experiments. In all experiments, the underlying proportional relationships were preserved while continuous perceptual magnitude cues were systematically altered. **(B)** Experiment 1: Unilateral enlargement. The cumulative area of one proportion stimulus was increased while its internal proportional structure remained unchanged, allowing us to test whether perceptual enlargement biases relational judgments. **(C)** Experiment 2: Unilateral reduction. The cumulative area of one proportion stimulus was reduced, providing a complementary test of whether perceptual influence reflects general sensitivity to continuous magnitude rather than enlargement-specific salience. **(D)** Experiment 3: Bidirectional congruent and incongruent scaling. The areas of the two competing proportion stimuli were simultaneously increased and decreased to create perceptual cues that either aligned with or conflicted with the underlying proportional relationship. Congruent and incongruent conditions were defined according to whether the manipulated perceptual magnitude preserved or violated the natural perceptual signatures associated with the proportional structure. **(E)** Experiment 4: Cue-strength manipulation. The magnitude of perceptual scaling was parametrically varied (2-fold vs. 5-fold) to examine whether the influence of perceptual cues increased monotonically with cue strength or exhibited bounded dynamics.

First, we investigate whether continuous perceptual information influences proportion judgments even when the underlying proportional structure is preserved (Gebuis and Reynvoet, 2012b; Leibovich et al., 2017). Experiments 1 and 2 manipulate perceptual magnitude unilaterally through area expansion and contraction, respectively, allowing us to determine whether perceptual effects depend on the direction of scaling or instead reflect a broader sensitivity to continuous visual information. Second, we examine how perceptual and relational information interact when they provide converging or conflicting evidence. Experiment 3 introduces bidirectional congruent and incongruent manipulations, enabling a direct comparison between conditions in which perceptual cues support or conflict with the underlying proportional relationship (Matthews and Lewis, 2017). Third, we test whether the influence of perceptual cues increases monotonically with cue strength or exhibits bounded dynamics. Experiment 4 parametrically manipulates the magnitude of area scaling, providing a direct examination of whether stronger perceptual signals produce progressively larger behavioral effects or whether their influence becomes constrained under extreme cue conditions (Ernst and Banks, 2002; Körding et al., 2007).

In addition to behavioral analyses, we implement hierarchical drift–diffusion modeling to characterize how relational and perceptual information contribute to evidence accumulation during proportion judgments (Ratcliff and McKoon, 2008; Wiecki et al., 2013). Specifically, we compare standard linear integration models with bounded integration models incorporating saturating transformations of perceptual input. This computational approach allows us to move beyond describing perceptual biases and to characterize the computational processes underlying the integration of relational and perceptual information.

Together, these experiments address a fundamental question in cognitive science: how abstract relational cognition remains grounded in perceptual systems while avoiding excessive reliance on extreme sensory signals.

## Experiment 1

The primary goal of Experiment 1 was to establish whether non-symbolic proportion judgments are susceptible to continuous perceptual magnitude information under conditions of minimal perceptual perturbation (DeWind et al., 2015). Specifically, we examined whether a unilateral manipulation of continuous perceptual signals – applied to only one of the two proportion stimuli – would systematically bias proportion comparison performance, despite leaving the underlying proportional structure unchanged.

This manipulation provides a sensitive test of the extent to which relational proportion representations remain influenced by continuous perceptual variation. According to strong interpretations of the Ratio Processing System (RPS), proportion judgments rely primarily on relational representations that are relatively robust to variation in continuous perceptual properties (Boyer et al., 2008; Matthews et al., 2016; McCrink and Wynn, 2007). Because unilateral area scaling preserves both numerosity and internal ratio structure, highly abstract ratio representations would predict reduced sensitivity to such transformations. Under this account, changing the physical size of one stimulus should have relatively limited effects on proportion discrimination.

By contrast, perceptual integration frameworks predict that even localized and task-irrelevant visual perturbations may penetrate relational comparison processes (Gebuis and Reynvoet, 2012a; Henik and Tzelgov, 1982; Leibovich et al., 2017). Continuous visual dimensions such as continuous perceptual signals are known to influence non-symbolic quantity judgments, even when they are formally irrelevant to the task (Gevers et al., 2016; Lourenco and Aulet, 2023). Importantly, unilateral manipulations allow us to test whether perceptual information contributes to evidence accumulation during proportion comparison, rather than merely reflecting global stimulus salience or generalized visual restructuring (DeWind et al., 2015).

A second objective of Experiment 1 was to examine whether the influence of perceptual information varies as a function of ratio distance. Ratio distance is a well-established determinant of discrimination difficulty in both numerosity and proportion processing (Jacob and Nieder, 2009a,2009b; Pinel et al., 2004). Rather than assuming that perceptual cues simply compensate for representational uncertainty, the present framework predicts that ratio distance may modulate the extent to which perceptual and relational information interact during decision formation. Thus, Experiment 1 provides an initial test of whether weak perceptual perturbations are sufficient to bias proportion judgments and whether such effects vary across levels of relational discriminability.

If proportion representations are largely independent of continuous perceptual variation, unilateral perceptual magnitude conditions should have minimal effects on performance. Conversely, if perceptual information contributes to relational decision processes, even weak unilateral manipulations should systematically bias accuracy and response time.

### Method

#### Transparency and openness

We report how we determined our sample sizes, all data exclusions, all manipulations, and all measures in the study, and we follow the Journal Article Reporting Standards (Appelbaum et al., 2018). All de-identified behavioral data, stimulus-generation materials, and analysis scripts for all four experiments have been made publicly available on the Open Science Framework (OSF) at the following anonymous link for peer review^1^. The repository includes scripts for data preprocessing, mixed-effects modeling, computational modeling, and figure generation. Upon acceptance, the anonymous link will be replaced with a permanent public DOI. The studies reported in this article were not preregistered.

Statistical analyses were conducted in R (Version 4.5.3) (R Core Team, 2023). Generalized and linear mixed-effects models were implemented using the lme4 package, and hierarchical Bayesian drift-diffusion modeling was implemented using brms (Bürkner, 2017). All participants provided informed consent prior to participation. The study protocol was approved by the Human Subjects Protection Committee of the School of Psychology at Guizhou Normal University.

### Participants

Thirty-six undergraduate students from a local university participated in the experiment (26 female; mean age = 20.61 years, range = 18–24). All participants reported normal or corrected-to-normal vision and provided written informed consent prior to participation. The study was conducted in accordance with the Declaration of Helsinki and was approved by the Institutional Review Board of the authors’ university.

An *a priori* sample size estimation was conducted using G*Power (Faul et al., 2007) based on a conservative repeated-measures design approximation. Sample size was determined using a conservative repeated-measures approximation implemented in G*Power. Assuming a medium within-subject effect (f = 0.25), α = 0.05, and desired power of 0.90, the analysis suggested that approximately 36 participants would provide adequate sensitivity for detecting theoretically meaningful effects. Because the primary analyses were conducted using trial-level mixed-effects models, these models allowed us to account for repeated observations within participants and trial-level variability (Baayen et al., 2008; Barr, 2013).

#### Design

The experiment employed a 2 (ratio distance: near, far) × 3 (perceptual magnitude manipulation: baseline, larger-proportions enlarged, smaller-proportions enlarged) within-subjects design. A total of eight unique non-symbolic proportion pairs served as the base stimuli (Table 1). These stimuli were combined across the experimental conditions to create 32 unique trial types. Each trial type was repeated six times, yielding 192 experimental trials per participant. Across repetitions, the spatial position of the larger proportion (left vs. right) was counterbalanced. Trials were divided into two blocks separated by a short rest break, and all trials were presented in a randomized order.

**TABLE 1:** Ratio sets used in all experiments.

Ratio distance	Proportion pairs
Near	1:3 vs. 2:7
	1:4 vs. 2:9
	7:8 vs. 5:6
	4:9 vs. 3:7
Far	7:8 vs. 2:9
	4:5 vs. 2:9
	4:9 vs. 1:8
	5:7 vs. 1:4

In the enlargement condition, the area of each individual circle in one proportion stimulus was uniformly scaled by a factor of two, thereby increasing the area of both subsets (numerator and denominator) without altering numerosity or the internal ratio. The other stimulus remained unchanged.

Critically, the perceptual magnitude condition was defined relative to the numerical magnitude of the compared proportions. In the larger-proportion enlarged condition, the cumulative area of the proportion with the numerically larger ratio was increased. In the smaller-proportion enlarged condition, the cumulative area of the numerically smaller proportion was increased. This manipulation allowed us to examine whether localized perceptual salience systematically alters relational comparison processes despite preserving proportional structure.

#### Stimuli

Stimuli consisted of pairs of non-symbolic proportions constructed from two vertically arranged circles (white and gray), representing the upper and lower components of each proportion. Each trial presented two proportion stimuli simultaneously on the screen. The top circle was white (RGB = 255, 255, 255) and the bottom circle was light gray (RGB = 180, 180, 180), presented against a dark gray background (RGB = 50, 50, 50). Eight base pairs were used across all four experiments (see Table 1), with the numbers representing relative area units. The pixel area was calculated by multiplying the relative area unit by 2800. The radius of each circle was determined using the formula (r$=\sqrt{\text{S}/\pi }$).

Stimuli were presented on a 1600 × 1200 pixel display. To minimize potential confounds arising from spatial separation, the edge-to-edge distance between the upper and lower circles within each proportion pair was held constant across all experimental conditions and trials. When circle area was enlarged or reduced, the vertical center-to-center distance between the circles was adjusted dynamically to preserve a constant inter-item gap (100 pixels). This procedure ensured that perceptual magnitude conditions did not introduce systematic changes in spacing or overlap. The same spacing-control procedure was later extended to the larger scaling manipulations used in Experiment 4. Unless otherwise specified, the same stimulus generation and positioning procedures were used across all four experiments.

The compared ratios differed numerically (e.g., 1:3 vs. 2:7), ensuring that one proportion was objectively larger than the other. The proportion pairs used in all experiments are presented in Table 1 and ratio distance was defined as the absolute numerical difference between the two compared proportions. The near-ratio condition consisted of proportion pairs with relatively small differences (mean ratio distance = 0.033). In contrast, the far-ratio condition consisted of proportion pairs with larger differences (mean ratio distance = 0.504). The selected ratio pairs therefore created clearly separable levels of relational discriminability, with near pairs requiring fine-grained proportional comparison and far pairs involving substantially larger relational differences.

To manipulate continuous perceptual magnitude, unilateral scaling was applied to one proportion stimulus. Specifically, all circles belonging to one proportion stimulus were uniformly enlarged by a factor of two, thereby increasing cumulative area while preserving numerosity and internal ratio structure. The comparison stimulus remained physically unchanged. In the larger-proportion enlarged condition, the larger proportion was enlarged. In the smaller-proportion enlarged condition, the smaller proportion was enlarged. In the baseline condition, neither stimulus was manipulated. Hence, single circles in the baseline condition ranged from 2,800 to 25,200 pixels. In the enlargement conditions, the two circles on one side (either the large or small proportion side) were doubled in area, resulting in a range of 5,600 to 50,400 pixels, while the other side remained unchanged.

Cumulative area was selected because it represents one of the most extensively studied continuous magnitude cues in the numerical cognition literature and has been shown to systematically influence quantity judgments independently of numerosity (Gebuis and Reynvoet, 2012a; Leibovich et al., 2017). Additionally, recent work emphasizes that effects of continuous magnitude cues depend on their perceptual discriminability and visual salience (Aulet and Lourenco, 2023a).

#### Procedure

Each trial began with a fixation cross presented for 500 ms, followed by a blank interval ranging from 250 to 300 ms. The stimulus pair was then presented for up to 1500 ms. Participants were instructed to indicate which stimulus represented the larger proportion value, defined by the relationship between the upper white circle and the lower gray circle within each stimulus by pressing the “F” key for the left stimulus or the “J” key for the right stimulus as quickly and accurately as possible. No performance feedback was provided. The spatial position of the larger proportion and the enlarged stimulus was counterbalanced across trials. Before the formal experiment, participants completed 16 practice trials to familiarize themselves with the task.

#### Dependent measures and data analysis

Accuracy data were analyzed using generalized linear mixed-effects models (GLMMs) with a binomial link function. Reaction times (RTs) were analyzed using linear mixed-effects models (LMMs) based on correct trials only. Trials with RTs shorter than 200 ms or exceeding 3 standard deviations above each participant’s mean were excluded prior to analysis. RTs were log-transformed to reduce positive skewness.

For both GLMMs and LMMs, fixed effects included ratio distance, perceptual magnitude condition, and their interaction. Random intercepts for participants were included in all models, and random slopes were included whenever model convergence permitted. Categorical predictors were effect-coded prior to analysis.

### Results

The raw dataset comprised 6,912 trials from 36 participants. For the accuracy analysis, all trials were retained; trials without a response (n = 112, 1.62%) were recorded as incorrect by the experimental program and therefore contributed to the accuracy measure. RT analyses were restricted to correct-response trials (n = 5,341). Of these, one trial with an RT shorter than 200 ms and 21 trials exceeding 3 SDs above the participant-specific mean were excluded, leaving 5,319 valid correct-RT observations for analysis. RTs were log-transformed prior to modeling. Detailed condition-level descriptive statistics, including observed accuracy, correct-trial RTs, and the number of valid observations, are reported in Supplementary Table 1.

#### Accuracy

Accuracy was analyzed using a generalized linear mixed-effects model (binomial link function), with ratio distance (near vs. far) and perceptual magnitude condition (baseline, large proportion enlargement, small proportion enlargement) specified as fixed effects, and participant-level random intercepts included to account for repeated measurements (Table 2).

**TABLE 2:** Generalized linear mixed-effects model results for accuracy.

Predictor	Estimate (β)	SE	*Z* values	*P* values
Intercept	0.243	0.068	3.564	<0.001
Ratio distance	3.025	0.167	18.165	<0.001
Large proportion enlargement (vs. baseline)	0.199	0.085	2.335	0.020
Small proportion enlargement (vs. baseline)	0.195	0.085	2.292	0.022
Ratio distance × large proportion enlargement	−0.827	0.212	−3.895	<0.001
Ratio distance × small proportion enlargement	−0.067	0.242	−0.275	0.78

Accuracy was analyzed using a generalized linear mixed-effects model (binomial logit link). Baseline and near-ratio conditions served as reference levels. Positive coefficients indicate higher log-odds of correct responses.

The model revealed a significant main effect of ratio distance, β = 3.03, *SE* = 0.17, *z* = 18.17, *p* < 0.001, indicating substantially higher accuracy for far than near ratios. This pattern is consistent with the well-established ratio-distance effect in non-symbolic proportion processing. Importantly, the effect of larger-proportion enlargement varied significantly as a function of ratio distance, as indicated by the ratio distance × larger-proportion enlargement interaction, β = −0.83, *SE* = 0.21, z = −3.90, *p* < 0.001. In contrast, the ratio distance × smaller-proportion enlargement interaction was not significant (Figure 2A).

**FIGURE 2:**
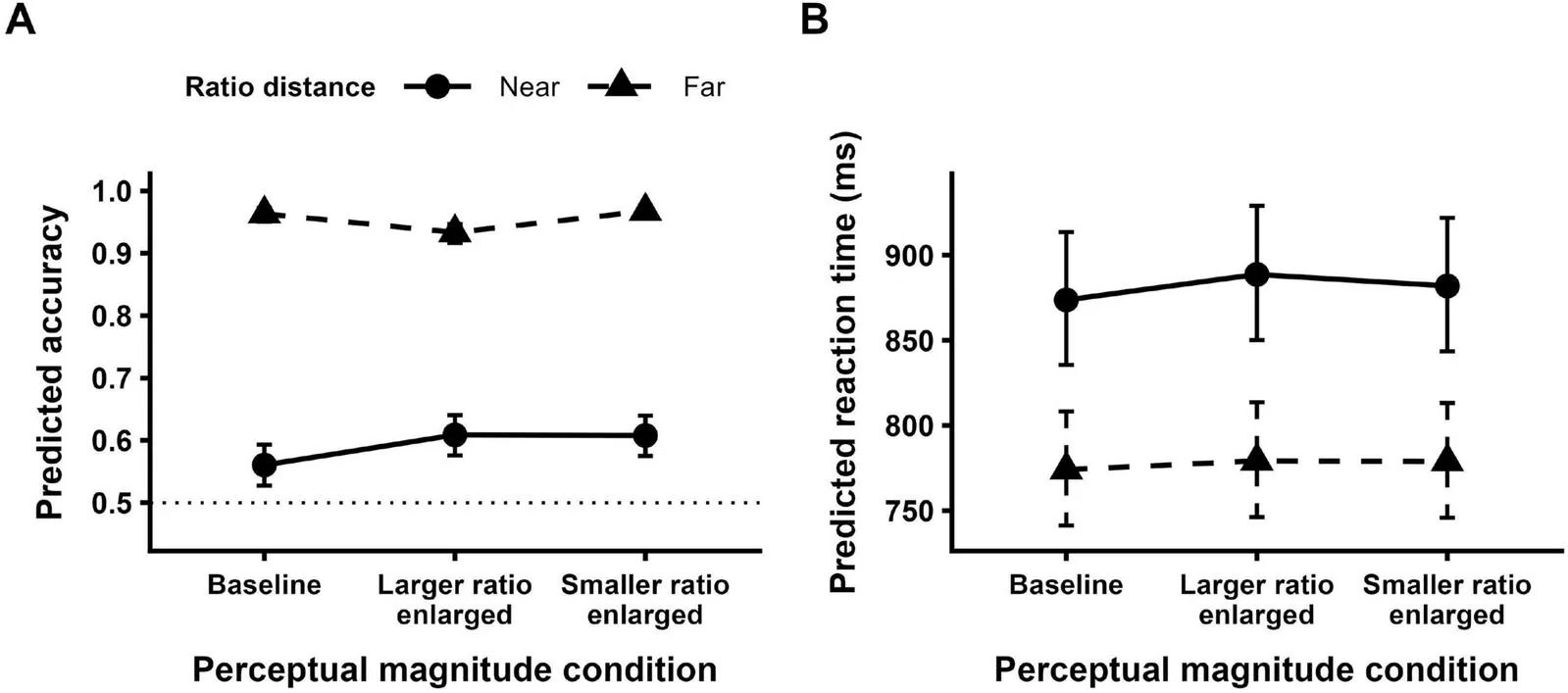
Effects of unilateral perceptual magnitude enlargement on non-symbolic proportion judgments in Experiment 1. **(A)** Shows model-estimated accuracy as a function of ratio distance and perceptual magnitude condition. The horizontal dotted line indicates chance-level performance (0.50). **(B)** Shows model-estimated reaction times for correct trials, back-transformed from the log scale to milliseconds for visualization. Error bars represent model-based 95% confidence intervals.

Follow-up comparisons further clarified this interaction pattern (see Supplementary Figure 1). Under near-ratio conditions, both enlargement manipulations were associated with modest increases in predicted accuracy relative to baseline. Enlarging the larger-proportion stimulus increased predicted accuracy by 4.8 percentage points (95% CI [0.2, 9.4], *p* = 0.037), whereas enlarging the smaller-proportion stimulus increased predicted accuracy by 4.7 percentage points (95%CI [0.2, 9.3], *p* = 0.041). Under far-ratio conditions, the pattern differed. Enlarging the larger-proportion stimulus reduced predicted accuracy by 3.0 percentage points relative to baseline (95% CI [−5.0, −1.0], *p* = 0.002), whereas enlarging the smaller-proportion stimulus produced little change relative to baseline (0.4 percentage points, 95% CI [−1.2, 2.1], *p* = 0.783). Thus, the effect of unilateral enlargement varied across ratio distances and depended on which proportion stimulus was enlarged.

Taken together, these results indicate that unilateral perceptual magnitude scaling did not produce a uniform behavioral bias. Instead, the pattern of perceptual influence differed across ratio distances: both enlargement manipulations were associated with modest facilitation under near-ratio conditions, whereas under far-ratio conditions, enlargement of the larger-proportion stimulus produced a selective reduction in accuracy while enlargement of the smaller-proportion stimulus had little effect.

#### Reaction time

Reaction time data (correct trials only) were analyzed using a linear mixed-effects model on log-transformed RTs, with the same fixed and random structure as the accuracy analysis (Table 3).

**TABLE 3:** Linear mixed-effects model results for log-transformed reaction time.

Predictor	Estimate (β)	SE	df	t value	*P* value
Intercept	6.774	0.023	46.50	299.73	< .001
Ratio distance	−0.122	0.011	5299	−11.06	<0.001
Large proportion enlargement (vs. baseline)	0.016	0.012	5300	1.33	0.184
Small proportion enlargement (vs. baseline)	0.011	0.012	5300	0.90	0.366
Ratio distance × large proportion enlargement	−0.009	0.015	5399	−0.57	0.568
Ratio distance × small proportion enlargement	−0.005	0.015	5300	−0.32	0.747

Reaction times were log-transformed prior to analysis. Linear mixed-effects models were fitted with Satterthwaite approximations for degrees of freedom. Baseline and near-ratio conditions served as reference levels. Negative coefficients indicate faster responses.

A significant main effect of ratio distance was observed, β = −0.122, *SE* = 0.011, *t* = −11.06, *p* < 0.001, indicating faster responses for far than near ratios. Neither enlargement condition differed significantly from baseline: larger-proportion enlargement, β = 0.016, *SE* = 0.012, *t* = 1.33, *p* = 0.184; smaller-proportion enlargement, β = 0.011, *SE* = 0.012, *t* = 0.90, *p* = 0.366. Moreover, neither the ratio distance × larger-proportion enlargement interaction, β = −0.009, *SE* = 0.015, *t* = −0.57, *p* = 0.568, nor the ratio distance × smaller-proportion enlargement interaction, β = −0.005, *SE* = 0.015, *t* = −0.32, *p* = 0.747, reached significance (Figure 2B).

Thus, although response times showed the expected ratio-distance effect, there was little evidence that unilateral enlargement reliably altered response speed. In Experiment 1, the effects associated with perceptual magnitude manipulation were therefore more evident in accuracy than in correct-trial RTs.

### Discussion

Experiment 1 provided initial evidence that unilateral enlargement of continuous perceptual magnitude can influence non-symbolic proportion judgments even when proportional relations remain unchanged. These effects depended on ratio distance and on which stimulus was manipulated. For near ratios, enlarging either the larger- or smaller-proportion stimulus produced modest facilitation relative to baseline. For far ratios, however, enlarging the larger-proportion stimulus reduced accuracy, whereas enlarging the smaller-proportion stimulus produced little reliable change. This context dependence is consistent with evidence that continuous visual properties can modulate numerical and proportional judgments despite being formally irrelevant to the target relation (DeWind et al., 2015; Leibovich et al., 2017; Matthews and Lewis, 2017).

The ratio-distance dependence suggests that perceptual information does not exert a fixed bias but interacts with the discriminability of relational evidence. Near ratios require finer proportional discrimination than far ratios (Park et al., 2021). The present pattern does not support a simple rule whereby less precise relational representations always increase reliance on perceptual information. Instead, the effects varied qualitatively across conditions, consistent with a context-sensitive perceptual–relational interaction (Ernst and Banks, 2002; Ernst and Bülthoff, 2004). The far-ratio accuracy cost observed when the larger-proportion stimulus was enlarged also represents a conditional asymmetry. Because participants were always instructed to select the larger proportion, this asymmetry should be interpreted within the current response frame rather than as evidence that the larger proportion is intrinsically more decision relevant.

Perceptual effects were more evident in accuracy than in correct-trial RTs, which showed a robust ratio-distance effect but no reliable enlargement effect. Because RT analyses were restricted to correct responses, this difference should be interpreted descriptively rather than as evidence for a specific processing stage (Ratcliff and McKoon, 2008; Wiecki et al., 2013).

Finally, Experiment 1 cannot determine whether the observed effects reflect sensitivity to continuous perceptual magnitude generally or properties specific to enlargement, such as increased salience or attentional priority (Carrasco, 2011). Experiment 2 therefore introduced the complementary manipulation of unilateral contraction to test whether similar effects would occur when perceptual magnitude was reduced rather than increased.

## Experiment 2

Experiment 1 provided initial evidence that unilateral enlargement of continuous perceptual magnitude can influence non-symbolic proportion judgments even when the underlying proportional relationships remain unchanged. Importantly, these effects were not uniform across conditions. Under near-ratio conditions, enlargement of either the larger- or smaller-proportion stimulus was associated with modest facilitation relative to baseline. Under far-ratio conditions, however, enlarging the stimulus representing the larger proportion produced a reliable reduction in accuracy, whereas enlarging the smaller-proportion stimulus produced little change. Thus, the behavioral consequences of perceptual scaling varied as a function of both ratio distance and which stimulus was manipulated, consistent with broader evidence that continuous visual properties can systematically modulate numerical and proportional judgments (Carrasco, 2011; DeWind et al., 2015; Leibovich et al., 2017; Matthews and Lewis, 2017; Treisman and Gelade, 1980).

Experiment 2 addressed this ambiguity using a complementary manipulation in which one proportion stimulus was contracted rather than enlarged. If the effects observed in Experiment 1 were driven primarily by enlargement-specific salience, reducing stimulus area should produce weaker or qualitatively different effects. In contrast, if continuous perceptual magnitude contributes to proportion comparison more generally, manipulating physical magnitude in the opposite direction should also systematically influence performance, despite preservation of the underlying proportional relationship (DeWind et al., 2015; Gebuis and Reynvoet, 2012a; Leibovich et al., 2017).

This complementary manipulation is important because it separates the direction of physical scaling from the broader question of perceptual-relational integration. The critical issue is not simply whether a stimulus becomes physically larger or smaller, but whether task-irrelevant changes in continuous perceptual magnitude alter a judgment that formally depends on the relationship between the two component areas. Evidence of systematic effects under contraction would therefore strengthen the conclusion that perceptual influences cannot be attributed solely to enlargement-related salience.

As in Experiment 1, we also examined whether the effect of perceptual magnitude manipulation varied with ratio distance. The same near- and far-ratio pairs were used, providing conditions of relatively lower and higher relational discriminability, respectively. Rather than assuming that perceptual cues compensate for an imprecise relational representation, we asked whether the contribution of perceptual information changes as a function of the discriminability of the underlying proportional relationship. If proportion judgments remain relatively invariant to continuous perceptual transformations, unilateral contraction should have limited effects on performance. Conversely, if relational and perceptual information jointly contribute to proportion comparison, systematic effects should also emerge when perceptual magnitude is reduced.

### Method

#### Participants

A new sample of 34 undergraduate students participated in Experiment 2 (29 female; mean age = 21.75 years, range = 18–25 years). All participants reported normal or corrected-to-normal vision and provided written informed consent prior to participation. The study was conducted in accordance with the Declaration of Helsinki and was approved by the Institutional Review Board of the authors’ university.

Sample size determination followed the same rationale as in Experiment 1. An *a priori* sample size estimation was conducted using G*Power (Faul et al., 2007), based on a conservative repeated-measures approximation. Assuming a medium-sized within-subject effect (f = 0.25), α = 0.05, and desired power = 0.90, the analysis indicated that approximately 34 participants would provide adequate sensitivity to detect theoretically meaningful effects. The primary analyses were subsequently conducted using trial-level GLMMs and LMMs to account for repeated observations and trial-level variability within participants (Baayen et al., 2008; Barr, 2013).

#### Design

Experiment 2 employed a 2 (ratio distance: near, far) × 3 (perceptual magnitude condition: baseline, larger-proportion contracted, smaller-proportion contracted) within-subjects design. The same eight base proportion pairs used in Experiment 1 served as stimuli (Table 1), yielding 32 unique trial types across conditions. Each trial type was repeated six times, resulting in 192 experimental trials per participant. Across repetitions, the left-right position of the stimulus with the objectively larger proportion value was counterbalanced. Trials were presented in randomized order and divided into two experimental blocks separated by a short break.

Experiment 2 differed from Experiment 1 specifically in the direction of perceptual scaling. Whereas Experiment 1 used unilateral enlargement, Experiment 2 used unilateral contraction. This complementary manipulation allowed us to examine whether perceptual influences were specific to enlargement or were also observed when continuous perceptual magnitude was reduced.

#### Stimuli

Stimuli were identical in overall format to those used in Experiment 1. Each proportion stimulus consisted of two vertically arranged filled circles: an upper white circle and a lower light-gray circle. The ratio between the areas of the upper and lower circles defined the proportional value of the stimulus. Two proportion stimuli were presented simultaneously, one on each side of the screen, and one stimulus represented an objectively larger proportion value than the other. Continuous perceptual magnitude was manipulated through unilateral contraction of one proportion stimulus. Specifically, the areas of both circles belonging to one stimulus were reduced to one-half of their original values. Because the same scaling factor was applied to both component circles, their area ratio – and therefore the proportional value represented by the stimulus – remained unchanged.

In the larger-proportion contracted condition, the stimulus representing the objectively larger proportion value was reduced while the stimulus representing the smaller proportion value remained physically unchanged. In the smaller-proportion contracted condition, the stimulus representing the objectively smaller proportion value was reduced while the larger-proportion stimulus remained unchanged. In the baseline condition, neither stimulus was scaled. The baseline single-circle areas ranged from 2,800 to 25,200 pixels. In the contraction conditions, the two component circles on the manipulated side were each reduced to one-half of their original area, resulting in a range of 1,400–12,600 pixels, while the comparison stimulus remained at its baseline size. Thus, the manipulation changed the absolute perceptual magnitude of one stimulus while preserving the proportional relationship between its upper and lower component areas.

This complementary contraction manipulation allowed us to assess whether the behavioral effects observed under enlargement in Experiment 1 could also be observed when perceptual magnitude was changed in the opposite physical direction, thereby reducing the likelihood that the effects were attributable solely to enlargement-related visual salience.

#### Procedure

The procedure was identical to that used in Experiment 1. Each trial began with a fixation cross presented for 500 ms, followed by a blank interval of 250–300 ms. The two proportion stimuli were then presented for up to 1500 ms. Participants judged which side represented the larger proportion value based on the area relationship between the upper white circle and the lower gray circle within each stimulus. They responded by pressing the “F” key for the left stimulus or the “J” key for the right stimulus as quickly and accurately as possible. No performance feedback was provided.

The experiment consisted of 192 experimental trials. The 32 unique trial types were each repeated six times, with the left–right position of the stimulus representing the larger proportion value counterbalanced across repetitions. Trials were presented in randomized order and divided into two blocks separated by a short break. Before the formal experiment, participants completed 16 practice trials to familiarize themselves with the task.

#### Dependent measures and data analysis

Data preprocessing and statistical analyses followed the same procedures as in Experiment 1. Accuracy was analyzed using generalized linear mixed-effects models (GLMMs) with a binomial link function. RTs were analyzed using linear mixed-effects models (LMMs) based on correct trials only. Trials with RTs shorter than 200 ms or exceeding 3 standard deviations above the participant-specific mean were excluded prior to analysis, and RTs were subsequently log-transformed to reduce positive skew. Fixed effects included ratio distance, perceptual magnitude condition, and their interaction. Participant-level random intercepts were included in all models, with random slopes added whenever supported by model convergence.

### Results

The dataset comprised 6,528 trials from 34 participants. Accuracy analyses were conducted on all trials. For the reaction-time analysis, 130 trials with RTs shorter than 200 ms and a further 25 trials exceeding three standard deviations above the participant-specific mean were excluded, resulting in 155 RT exclusions (2.37% of all trials). RT analyses were subsequently restricted to correct responses, yielding 4,387 valid correct-RT observations. Condition-level descriptive statistics and the numbers of valid correct-RT observations are reported in Supplementary Table 2.

#### Accuracy

Accuracy was analyzed using a generalized linear mixed-effects model with a binomial distribution and logit link function. Ratio distance (near vs. far), perceptual magnitude condition (baseline, smaller-proportion contracted, larger-proportion contracted), and their interaction were included as fixed effects, with participant-specific random intercepts to account for repeated observations (Table 4).

**TABLE 4:** Fixed effects of the generalized linear mixed-effects model for accuracy.

Predictor	Estimate (β)	SE	*Z* values	*P* values
Intercept	0.220	0.070	3.13	0.002
Ratio distance (far)	3.203	0.183	17.55	<0.001
Perceptual magnitude condition (smaller-proportion contracted)	0.249	0.088	2.84	0.005
Perceptual magnitude condition (larger-proportion contracted)	−0.287	0.086	−3.32	0.001
Ratio distance (far) × small proportion contracted	−0.721	0.237	−3.04	0.002
Ratio distance (far) × large proportion contracted	−3.233	0.202	−16.02	<0.001

Accuracy was modeled using a binomial distribution with a logit link function. The intercept represents the log-odds of a correct response for the reference condition (near ratio distance and baseline perceptual magnitude condition). Ratio distance was dummy-coded with Near as the reference level, and perceptual magnitude condition was dummy-coded with Baseline as the reference level. β = unstandardized estimate; SE = standard error.

The analysis revealed a robust main effect of ratio distance, β = 3.203, SE = 0.183, z = 17.550, *p* < 0.001, indicating substantially higher accuracy for far than near ratios. This pattern is consistent with the well-established ratio-distance effect in non-symbolic proportion processing. Importantly, the effects of unilateral contraction varied according to both ratio distance and which proportion stimulus was manipulated. At the near-ratio reference level, contracting the smaller-proportion stimulus was associated with higher accuracy than baseline, β = 0.249, SE = 0.088, z = 2.839, *p* = 0.005, whereas contracting the larger-proportion stimulus was associated with lower accuracy, β = −0.287, SE = 0.086, z = −3.317, *p* = 0.001. Moreover, both effects varied significantly with ratio distance: ratio distance × smaller-proportion contraction, β = −0.721, SE = 0.237, z = −3.04, *p* = 0.002; ratio distance × larger-proportion contraction, β = −3.233, SE = 0.202, z = −16.02, *p* < 0.001.

Follow-up comparisons against baseline further clarified these interactions (see Supplementary Figure 2). Under near-ratio conditions, contracting the smaller-proportion stimulus increased predicted accuracy by approximately 6.0 percentage points, 95%CI [1.3, 10.7], Dunnett-adjusted *p* = 0.009, whereas contracting the larger-proportion stimulus reduced predicted accuracy by approximately 7.1 percentage points, 95% CI [−11.9, −2.4], *p* = 0.002. Under far-ratio conditions, smaller-proportion contraction produced only a modest reduction of approximately 1.8 percentage points relative to baseline, 95% CI [−3.7, 0.1], *p* = 0.059, whereas larger-proportion contraction produced a pronounced reduction of approximately 49.3 percentage points, 95% CI [−53.2, −45.3], *p* < 0.001.

The corresponding model-estimated accuracies were 0.555 for baseline, 0.615 for smaller-proportion contraction, and 0.483 for larger-proportion contraction under near-ratio conditions. Under far-ratio conditions, estimated accuracy was 0.968 for baseline, 0.950 for smaller-proportion contraction, and 0.476 for larger-proportion contraction (Figure 3A). Thus, performance in the far-ratio larger-proportion-contraction condition was reduced to approximately chance level, whereas baseline and smaller-proportion-contraction performance remained near ceiling.

**FIGURE 3:**
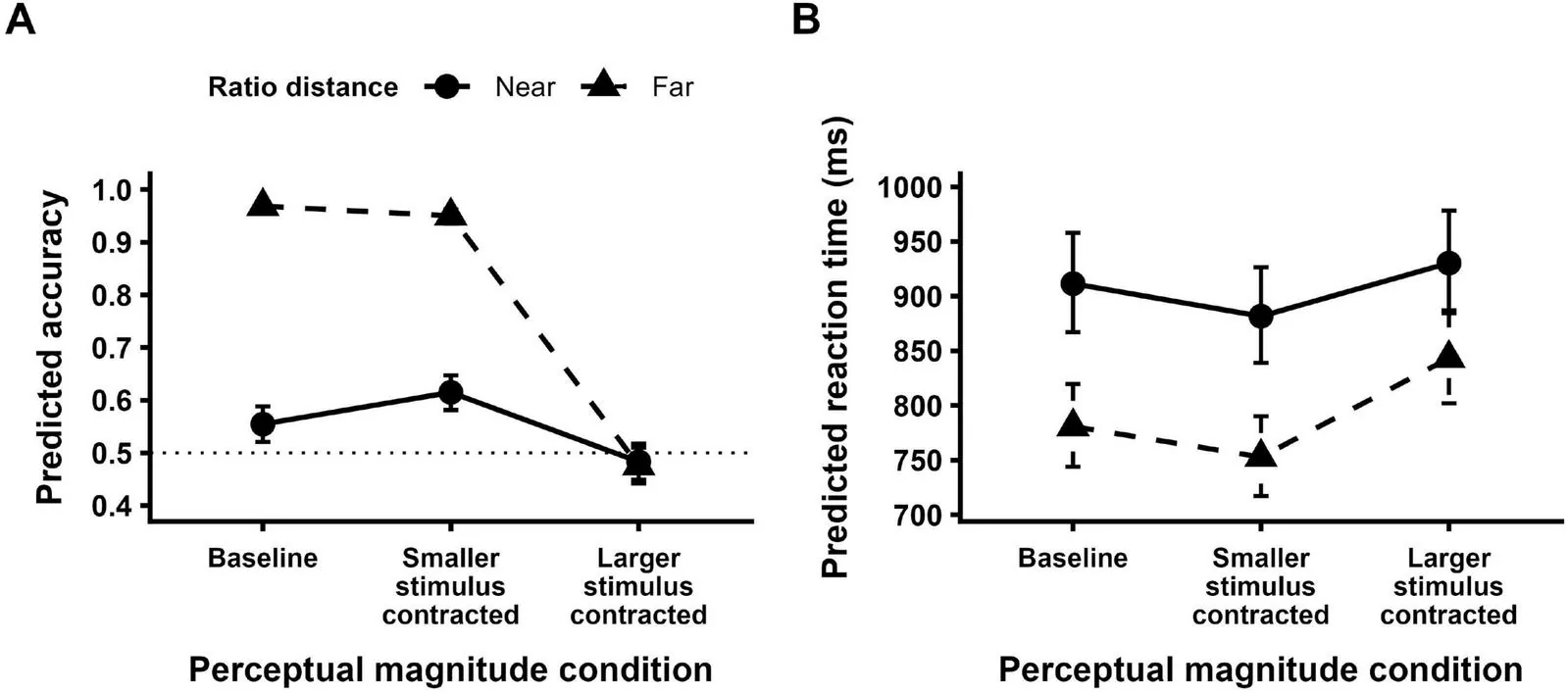
Effects of unilateral perceptual magnitude contraction on non-symbolic proportion judgments in Experiment 2. **(A)** Shows model-estimated accuracy as a function of ratio distance and perceptual magnitude condition. The horizontal dotted line indicates chance-level performance (0.50). **(B)** Shows model-estimated reaction times for correct trials, back-transformed from the log scale to milliseconds for visualization. Error bars represent model-based 95% confidence intervals.

Taken together, unilateral contraction did not exert a uniform effect on proportion judgments. Its behavioral consequences depended strongly on both ratio distance and which of the two proportion stimuli was contracted.

#### Reaction time

Reaction times for correct trials were log-transformed and analyzed using a linear mixed-effects model with the same fixed- and random-effects structure as the accuracy model (Table 5).

**TABLE 5:** Fixed effects of the linear mixed-effects model for log-transformed reaction time.

Predictor	Estimate (β)	SE	df	*t* value	*P* value
Intercept	6.815	0.025	42.64	268.11	<0.001
Ratio distance (far)	−0.155	0.011	4348.51	−13.59	<0.001
perceptual magnitude condition (smaller proportion contracted)	−0.033	0.013	4348.95	−2.65	0.008
perceptual magnitude condition (larger proportion contracted)	0.020	0.013	4350.04	1.53	0.126
Ratio distance (far) × smaller proportion contracted	−0.003	0.016	4348.55	−0.21	0.835
Ratio distance (far) × larger proportion contracted	0.057	0.018	4348,97	3.15	0.002

Reaction times for correct trials were log-transformed prior to analysis. The intercept represents the predicted mean log-transformed RT for the reference condition (near ratio distance and baseline perceptual magnitude condition). Ratio distance was dummy-coded with Near as the reference level, and perceptual magnitude condition was dummy-coded with Baseline as the reference level. Degrees of freedom were estimated using Satterthwaite’s approximation. β = unstandardized estimate; SE = standard error.

The model revealed a significant effect of ratio distance, β = −0.155, SE = 0.011, t = −13.59, *p* < 0.001, indicating faster responses for far than near ratios. At the near-ratio reference level, responses were significantly faster when the smaller-proportion stimulus was contracted than in the baseline condition, β = −0.033, SE = 0.013, t = −2.65, *p* = 0.008. In contrast, larger-proportion contraction did not significantly differ from baseline, β = 0.020, SE = 0.013, t = 1.53, *p* = 0.126.

The ratio distance × smaller-proportion contraction interaction was not significant, β = −0.003, SE = 0.016, *t* = −0.21, *p* = 0.835. In contrast, the ratio distance × larger-proportion contraction interaction was significant, β = 0.057, SE = 0.018, *t* = 3.15, *p* = 0.002, indicating that the RT effect associated with larger-proportion contraction varied across ratio distances.

Model-estimated RTs further illustrated this pattern (Figure 3B). Under near-ratio conditions, estimated RTs were 911 ms for baseline, 882 ms for smaller-proportion contraction, and 930 ms for larger-proportion contraction. Under far-ratio conditions, the corresponding estimates were 781, 753, and 843 ms, respectively. Thus, responses were descriptively fastest in the smaller-proportion-contraction condition and slowest in the larger-proportion-contraction condition at both ratio distances, with the separation involving larger-proportion contraction being more pronounced for far ratios.

These RT results should be interpreted cautiously because the analysis was restricted to correct trials. This issue is particularly relevant for the far-ratio larger-proportion-contraction condition, in which accuracy was approximately at chance and substantially fewer trials contributed to the RT analysis than in the other far-ratio conditions. Accordingly, the RT findings are best treated as complementary to the accuracy results rather than as independent evidence for a specific processing mechanism.

### Discussion

Experiment 2 further showed that unilateral contraction of continuous perceptual magnitude can alter non-symbolic proportion judgments while leaving the underlying proportional relationships unchanged. These effects depended on both ratio distance and which stimulus was manipulated. Under near-ratio conditions, contracting the smaller-proportion stimulus modestly improved accuracy, whereas contracting the larger-proportion stimulus reduced it. Under far-ratio conditions, smaller-proportion contraction produced little reliable change, whereas larger-proportion contraction markedly reduced accuracy to approximately chance level. Thus, contraction did not produce a uniform bias but instead interacted with the relational context of the comparison.

This pattern is consistent with previous evidence that continuous visual properties can influence numerical and proportional judgments despite being formally irrelevant to the target relation (Gebuis and Reynvoet, 2012a; Leibovich et al., 2017; Matthews and Lewis, 2017). The strong dependence of the contraction effect on ratio distance also indicates that perceptual influence is context sensitive rather than fixed, broadly consistent with cue-integration accounts in which the contribution of perceptual information varies with the structure and reliability of available evidence (Ernst and Banks, 2002; Ernst and Bülthoff, 2004). The RT findings were generally compatible with this pattern, although they should be interpreted cautiously because analyses were restricted to correct trials, particularly in the low-accuracy far-ratio larger-contraction condition.

Together with Experiment 1, these findings show that perceptual effects are not limited to enlargement-specific salience, because systematic changes were also observed under contraction. However, because participants were always required to select the larger proportion, the observed asymmetries should be interpreted within this response frame rather than as evidence that one alternative is intrinsically more decision relevant. Moreover, both experiments manipulated only one member of the stimulus pair, creating a relative perceptual imbalance between the alternatives. Experiment 3 therefore introduced bidirectional scaling to examine perceptual–relational consistency more directly.

## Experiment 3

Experiments 1 and 2 demonstrated that manipulating continuous perceptual magnitude can systematically influence non-symbolic proportion judgments even when the underlying proportional relationships remain unchanged. These findings indicate that perceptual information contributes to proportion judgments beyond the relational structure specified by the proportions themselves. However, the unilateral manipulations used in the previous experiments altered only one of the competing proportion stimuli, providing limited information about how perceptual and relational information interact when they jointly contribute to the same decision.

Experiment 3 was designed to investigate how proportion judgments are affected when continuous perceptual magnitude cues either support or conflict with relational information. To achieve this goal, we introduced bidirectional perceptual magnitude manipulations, in which one proportion stimulus was enlarged while the other was simultaneously reduced. This manipulation increased the perceptual contrast between the two alternatives while preserving their underlying proportional relationships. By creating conditions in which perceptual and relational information were either aligned or in conflict, Experiment 3 allowed us to examine the consequences of perceptual facilitation and perceptual interference within a unified framework.

In the present experiment, perceptual congruity was defined according to whether the manipulated continuous perceptual magnitude provided evidence consistent with or conflicting with the relational structure required for the judgment. Importantly, perceptual congruity does not refer to a simple correspondence between physical size and proportional magnitude. Rather, it reflects whether the altered perceptual information supports or competes with the relational information needed to identify the larger proportion. In the congruent condition, the objectively larger proportion stimulus was reduced while the smaller proportion stimulus was enlarged, generating perceptual cues that converged with the relational judgment. In contrast, in the incongruent condition, the objectively larger proportion stimulus was enlarged while the smaller proportion stimulus was reduced, creating competing perceptual information that conflicted with the underlying relational structure.

This manipulation provides a direct test of competing accounts of non-symbolic proportion processing. If proportion judgments rely primarily on abstract relational representations that remain relatively stable across perceptual transformations, congruity should exert limited effects on performance. In contrast, perceptual integration accounts predict that relational judgments emerge from the combination of multiple sources of evidence, such that perceptually congruent information should facilitate decision making whereas incongruent information should impair performance (Boyer and Levine, 2012; Ernst and Banks, 2002; Gebuis et al., 2016; Gebuis and Reynvoet, 2012a; Leibovich et al., 2017; Lourenco and Aulet, 2023; Matthews and Lewis, 2017). Thus, observing systematic congruity effects would provide further evidence that perceptual information contributes to proportion judgments even when it is irrelevant to the instructed task.

Beyond examining whether perceptual cues influence proportion judgments, Experiment 3 also addressed whether facilitation and interference represent symmetric consequences of the same integration mechanism. Standard cue integration accounts suggest that congruent and incongruent perceptual cues reflect opposite changes in evidence weighting. However, theories of cognitive conflict and evidence accumulation suggest that conflicting information may impose additional processing demands beyond the simple absence of supportive evidence (Botvinick et al., 2001; Gold and Shadlen, 2007; Shenhav et al., 2013). Therefore, incongruent perceptual cues may produce stronger behavioral costs than congruent cues produce benefits, reflecting additional conflict resolution or suppression processes.

Accordingly, Experiment 3 tested two related predictions. First, if perceptual and relational information are jointly integrated during proportion judgments, congruent conditions should improve performance whereas incongruent conditions should impair performance relative to baseline. Second, if perceptual conflict recruits additional control mechanisms, incongruent interference may exceed congruent facilitation, resulting in asymmetric congruity effects.

### Method

#### Participants

Thirty-five undergraduate students participated in Experiment 3 (31 female; mean age = 20.69 years, range = 19–24 years). All participants reported normal or corrected-to-normal vision and provided written informed consent prior to participation. The study was conducted in accordance with the Declaration of Helsinki and approved by the Institutional Review Board of the authors’ university.

Sample size determination followed the same rationale as in the previous experiments. An *a priori* estimation conducted using G*Power indicated that approximately 34 participants would provide sufficient sensitivity to detect medium-sized within-subject effects under conservative assumptions. As in the previous experiments, the primary analyses were conducted using trial-level GLMMs and LMMs, which provide superior handling of repeated-measures variability relative to aggregate-based ANOVA approaches (Baayen et al., 2008; Barr, 2013).

#### Design

Experiment 3 employed a 2 (ratio distance: near, far) × 3 (perceptual congruity: baseline, congruent, incongruent) within-subjects design. The same eight base proportion pairs used in Experiments 1 and 2 served as stimuli (Table 1). Unlike the unilateral manipulations used in Experiments 1 and 2, Experiment 3 implemented bidirectional perceptual magnitude scaling. Specifically, the two competing proportion stimuli were simultaneously modified in opposite directions: one stimulus was enlarged while the other was reduced. This manipulation increased perceptual contrast between the alternatives while preserving the relational structure required for proportion comparison.

In the congruent condition, perceptual magnitude cues were manipulated to provide information consistent with the relational judgment required by the task. In the incongruent condition, the same scaling procedure was reversed, creating perceptual cues that conflicted with the relational information. The baseline condition preserved the original stimulus configuration without perceptual magnitude manipulation.

#### Stimuli

Stimuli were identical in overall format to those used in the previous experiments. Each stimulus consisted of two vertically arranged filled circles: an upper white circle and a lower gray circle. The ratio between their areas defined the proportional value of the stimulus. The upper circle was presented in white and the lower circle in gray, and their relative areas defined the proportional value of the stimulus. Two proportion stimuli were presented simultaneously on each trial. Participants were instructed to judge which side contained the stimulus with the larger proportion, based on the relationship between the upper white circle and the lower gray circle.

The congruent and incongruent conditions were defined according to whether the manipulated continuous perceptual magnitude cues were consistent with or conflicting with the underlying proportional relationship. Importantly, congruity did not refer to a direct correspondence between physical size and proportional magnitude. Instead, it reflected whether the altered perceptual information provided supportive or competing evidence for the relational judgment.

In the congruent condition, the objectively larger proportion stimulus was reduced by a factor of 2, whereas the smaller proportion stimulus was simultaneously enlarged by a factor of 2. This manipulation preserved the original proportional values while creating perceptual magnitude cues that converged with the relational information required for identifying the larger proportion. In the incongruent condition, the transformation was reversed: the objectively larger proportion stimulus was enlarged by a factor of 2, whereas the smaller proportion stimulus was reduced by a factor of 2. Although the proportional structure remained unchanged, this manipulation introduced perceptual magnitude cues that competed with the relational information. In the baseline condition, neither proportion stimulus was manipulated. The original area range was maintained (2,800-25,200 pixels). In both manipulated conditions, one stimulus was enlarged by a factor of 2 while the other was reduced to half of its original area, resulting in a single-circle area range of 1,400–50,400 pixels.

Importantly, these manipulations altered continuous perceptual magnitude while preserving the underlying proportional relationship between the two component areas within each stimulus. The upper white circle and the lower gray circle jointly defined the proportional value of each stimulus, and participants were instructed to judge which stimulus represented the larger proportion value while ignoring irrelevant changes in absolute physical size.

#### Procedure

The procedure was identical to that used in the previous experiments. Each trial began with a fixation cross presented for 500 ms, followed by a blank interval of 250-300 ms. The stimulus pair was then presented for 1500 ms. Participants judged which stimulus represented the larger proportion value by comparing the relative area relationship between the upper white circle and the lower gray circle within each stimulus. They responded by pressing the “F” key for the left stimulus or the “J” key for the right stimulus as quickly and accurately as possible. No feedback was provided.

The experiment consisted of 192 experimental trials. The 32 unique trial types generated in the experimental design were each repeated six times. The spatial position of the stimulus with the larger proportion value was counterbalanced across repetitions. Trials were presented in random order and divided into two experimental blocks separated by a short break. Prior to the formal experiment, participants completed 16 practice trials.

#### Dependent measures and data analysis

Data preprocessing and statistical analyses were identical to those used in the previous experiments. Accuracy was analyzed using GLMMs with a binomial link function. RTs for correct trials were analyzed using LMMs. Trials with RTs below 200 ms or exceeding 3 standard deviations above the participant-specific mean were excluded prior to analysis. RTs were subsequently log-transformed to reduce positive skew.

Fixed effects included ratio distance, congruity condition, and their interaction. Random intercepts for participants were included in all models, with random slopes added whenever supported by model convergence.

#### Computational modeling

To further characterize the mechanisms underlying congruity effects in non-symbolic proportion judgments, trial-level data from Experiment 3 were analyzed using a hierarchical drift-diffusion model (DDM). Whereas the behavioral analyses establish congruity effects at the level of accuracy and response time, the DDM provides a process-level account by decomposing performance into latent evidence accumulation dynamics (Ratcliff and McKoon, 2008; Wiecki et al., 2013).

Drift rate (v ) was modeled as a joint function of relational and perceptual signals, including an interaction between perceptual evidence and congruity condition. Relational signals were computed from the log-transformed ratio difference between the two stimuli, whereas perceptual signals were derived from the log-transformed continuous perceptual signals difference. This parameterization allowed perceptual contributions to evidence accumulation to vary as a function of perceptual congruity.

Only drift rate was allowed to vary as a function of experimental predictors. Boundary separation, starting point bias, and non-decision time were estimated hierarchically at the participant level but were not condition-specific. Models were estimated within a hierarchical Bayesian framework using the Wiener diffusion implementation in brms (Bürkner, 2017).

### Results

Accuracy and reaction-time analyses used separate exclusion procedures. For the accuracy analysis, 140 no-response trials (RT = 0; 2.03% of 6,912 trials) were excluded, leaving 6,772 trials. For the RT analysis, an additional 20 trials exceeding three standard deviations above each participant’s mean RT were removed, leaving 6,752 RT-valid trials. RT analyses were then restricted to correct responses, resulting in 5,154 observations. Condition-level descriptive statistics and valid correct-RT counts are reported in Supplementary Table 3.

#### Accuracy

Accuracy was analyzed using a generalized linear mixed-effects model with a binomial distribution and logit link function. Ratio distance (near vs. far), perceptual–relational condition (baseline, congruent, incongruent), and their interaction were included as fixed effects, with participant-specific random intercepts (Table 6).

**TABLE 6:** Generalized linear mixed-effects model results for accuracy.

Predictor	Estimate (β)	SE	z	p
Intercept	0.417	0.079	5.29	<0.001
Ratio distance (far)	2.515	0.145	17.32	<0.001
Incongruity vs. baseline	−0.531	0.087	−6.13	<0.001
Congruity vs. baseline	0.708	0.093	7.64	<0.001
Far × Incongruity	−0.393	0.181	−2.17	0.30
Far × congruity	0.661	0.208	−3.17	0.002

Near-ratio and baseline conditions served as reference levels. Estimates represent changes in log-odds of a correct response relative to the reference condition. β = unstandardized estimate; SE = standard error.

The model revealed a significant effect of ratio distance, β = 2.515, *SE* = 0.145, *z* = 17.32, *p* < 0.001, indicating substantially higher accuracy for far than near ratios. At the near-ratio reference level, incongruent trials were associated with lower accuracy than baseline, β = −0.531, SE = 0.087, *z* = −6.13, *p* < 0.001, whereas congruent trials were associated with higher accuracy, β = 0.708, SE = 0.093, z = 7.64, *p* < 0.001. Both effects varied significantly with ratio distance, as indicated by the ratio distance × incongruent interaction, β = −0.393, SE = 0.181, z = −2.17, *p* = 0.030, and the ratio distance × congruent interaction, β = −0.661, SE = 0.208, z = −3.17, *p* = 0.002.

Follow-up comparisons against baseline clarified these interactions (see Supplementary Figure 3). Under near-ratio conditions, incongruent trials reduced predicted accuracy by 13.1 percentage points, 95% CI [−17.8, −8.4], Dunnett-adjusted *p* < 0.001, whereas congruent trials increased accuracy by 15.2 percentage points, 95% CI [10.8, 19.6], *p* < 0.001. Under far-ratio conditions, incongruent trials remained associated with a reliable reduction in accuracy of 6.8 percentage points, 95% CI [−9.4, −4.2], *p* < 0.001. In contrast, congruent trials differed negligibly from baseline (0.2 percentage points, 95% CI [−1.7, 2.2], *p* = 0.943).

Model-estimated accuracies were 0.603, 0.755, and 0.471 for the baseline, congruent, and incongruent conditions, respectively, under near-ratio conditions. The corresponding estimates under far-ratio conditions were 0.949, 0.952, and 0.882 (Figure 4A). Thus, congruent information was associated with a pronounced accuracy benefit for near ratios but not for far ratios, whereas incongruent information reduced accuracy at both ratio distances.

**FIGURE 4:**
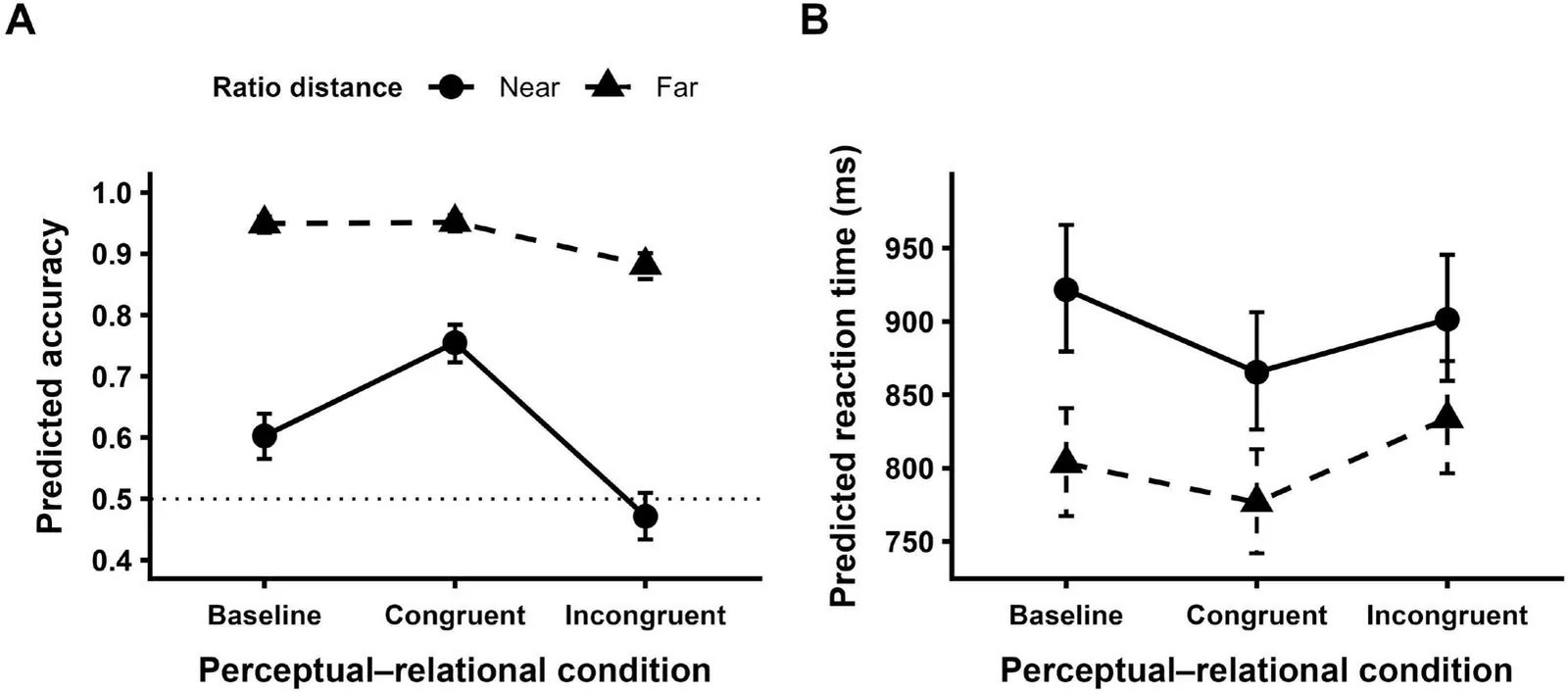
Effects of perceptual-relational congruity on non-symbolic proportion judgments in Experiment 3. **(A)** Shows model-estimated accuracy as a function of ratio distance and perceptual–relational condition. The horizontal dotted line indicates chance-level performance (0.50). **(B)** Shows model-estimated reaction times for correct trials, back-transformed from the log scale to milliseconds for visualization. Error bars represent model-based 95% confidence intervals.

#### Reaction time

RTs for correct trials were log-transformed and analyzed using a linear mixed-effects model with the same fixed-effects structure and participant-specific random intercepts (Table 7).

**TABLE 7:** Linear mixed-effects model results for log-transformed reaction time.

Predictor	Estimate (β)	SE	df	t	*p*
Intercept	6.826	0.024	42.51	285.87	<0.001
Ratio distance (far)	−0.138	0.011	5114.53	−13.00	<0.001
Incongruity vs. baseline	−0.022	0.013	5115.48	−1.76	0.078
Congruity vs. baseline	−0.063	0.011	5114.71	−5.67	<0.001
Far × Incongruity	0.06	0.016	5114.77	3.79	<0.001
Far × Congruity	0.029	0.014	5114.40	2.03	0.042

Reaction times were log-transformed prior to analysis. Near-ratio and baseline conditions served as reference levels. Degrees of freedom were estimated using Satterthwaite’s approximation. Negative coefficients indicate shorter log-transformed RTs relative to the reference condition.

The model revealed a significant effect of ratio distance, β = −0.138, SE = 0.011, *t* = −13.00, *p* < 0.001, indicating faster responses for far than near ratios. At the near-ratio reference level, incongruent trials did not differ reliably from baseline, β = −0.022, SE = 0.013, *t* = −1.76, *p* = 0.078, whereas congruent trials were associated with significantly faster responses, β = −0.063, SE = 0.011, *t* = −5.67, *p* < 0.001. Both condition effects varied with ratio distance: the ratio distance × incongruent interaction was significant, β = 0.060, SE = 0.016, *t* = 3.79, *p* < 0.001, as was the ratio distance × congruent interaction, β = 0.029, SE = 0.014, *t* = 2.03, *p* = 0.042.

Follow-up comparisons further clarified the RT pattern (see Supplementary Figure 3). Under near-ratio conditions, incongruent trials did not differ significantly from baseline (*p* = 0.142), whereas congruent trials were significantly faster (*p* < 0.001). Under far-ratio conditions, incongruent trials were significantly slower than baseline (*p* < 0.001), whereas congruent trials remained significantly faster (*p* < 0.001). Model-estimated RTs were 922, 865, and 901 ms for baseline, congruent, and incongruent trials, respectively, under near-ratio conditions, and 803, 777, and 834 ms under far-ratio conditions (Figure 4B).

Overall, the accuracy and RT results showed that the behavioral consequences of perceptual–relational congruity varied with ratio distance. Congruent information produced clear facilitation for near ratios in both accuracy and RT, whereas its accuracy benefit was absent for far ratios despite a remaining RT advantage. Incongruent information reduced accuracy at both ratio distances but produced reliable RT slowing only for far ratios. Because RT analyses were restricted to correct trials, these RT effects should be interpreted as complementary to the accuracy results rather than as independent evidence for a specific processing mechanism.

#### Computational modeling

Because facilitative and interfering effects were expressed differently across ratio distances and behavioral measures, accuracy and RT alone cannot determine how perceptual and relational information contribute to decision formation. To further characterize the computational structure underlying these effects, we therefore applied hierarchical drift-diffusion modeling to the trial-level data from Experiment 3. The model jointly characterized accuracy and RT to examine how relational and perceptual information contributed to evidence accumulation. Detailed model specification, parameter estimation, and cross-experiment comparisons are reported in the Computational Modeling section.

### Discussion

Experiment 3 showed that the effects of bidirectional perceptual magnitude manipulation depended on perceptual–relational congruity and ratio distance. Under near-ratio conditions, congruent information improved accuracy and accelerated responses, whereas incongruent information reduced accuracy. Under far-ratio conditions, incongruent information continued to impair accuracy and additionally slowed responses, whereas congruent information retained an RT advantage but no longer produced a detectable accuracy benefit. Thus, perceptual–relational congruity influenced performance in a context-dependent manner rather than producing a uniform behavioral effect.

These findings extend previous evidence that continuous visual properties influence numerical and proportional judgments by showing that facilitative and interfering perceptual effects were not simple mirror images (DeWind et al., 2015; Gevers et al., 2016; Leibovich et al., 2017). The congruent accuracy benefit was pronounced for near ratios but absent for far ratios, whereas the incongruent accuracy cost remained reliable at both ratio distances. The RT pattern showed a related asymmetry, with congruent information facilitating responding across ratio distances but reliable incongruent slowing emerging only for far ratios. This pattern is therefore better characterized as a context-dependent asymmetry in perceptual–relational effects than as a uniform integration bias. The absence of a far-ratio congruent accuracy benefit should nevertheless be interpreted cautiously because baseline performance was already near ceiling, and RT results were based on correct trials only.

Experiment 3 manipulated perceptual–relational alignment while holding scaling magnitude constant, leaving unresolved how perceptual influence changes as cue strength increases. Experiment 4 therefore compared moderate (2-fold) and stronger (5-fold) scaling to test whether increasing perceptual magnitude produces progressively larger behavioral effects or whether its contribution becomes functionally constrained.

## Experiment 4

Experiment 3 provided evidence that bidirectional perceptual magnitude manipulations influence non-symbolic proportion judgments depending on their consistency with relational information (Gebuis et al., 2016; Leibovich et al., 2017; Lourenco and Aulet, 2023; Matthews and Lewis, 2017). However, an important unresolved question concerns the functional relationship between perceptual cue strength and behavioral influence. Specifically, does the contribution of perceptual information increase continuously as cue strength increases, or is this contribution constrained under conditions of highly salient perceptual input?

Classical cue-integration frameworks predict that stronger perceptual cues should exert greater influence on behavior because cue weighting increases with signal reliability (Ernst and Banks, 2002; Ernst and Bülthoff, 2004). Under this account, increasing perceptual cue magnitude should produce progressively larger congruity effects, reflected in enhanced facilitation when cues support relational judgments and enhanced interference when cues conflict with them.

In contrast, bounded integration accounts propose that perceptual influence may not increase indefinitely. Instead, extremely strong perceptual cues may be subject to normalization-like constraints or reduced weighting due to contextual and ecological considerations (Carandini and Heeger, 2011; Körding et al., 2007; Körding and Wolpert, 2004; Summerfield and Tsetsos, 2015). Under this perspective, increasing cue magnitude beyond a moderate level may produce diminishing behavioral effects, suggesting a constrained rather than purely linear relationship between perceptual strength and decision influence.

Experiment 4 examined these competing predictions by parametrically manipulating the magnitude of bidirectional perceptual scaling. Building on the congruity manipulation introduced in Experiment 3, we contrasted a moderate scaling condition (2-fold transformation) with a stronger scaling condition (5-fold transformation). Importantly, scaling magnitude was manipulated proportionally rather than additively, allowing us to examine whether increasing perceptual magnitude produces corresponding increases in behavioral influence (Halberda et al., 2008; Piazza et al., 2004).

The present design therefore allows us to examine whether perceptual influence follows a monotonic relationship with cue strength or shows evidence of bounded dynamics. If perceptual integration is approximately linear, stronger scaling should produce larger congruity effects. Conversely, if perceptual integration is constrained, increasing cue magnitude beyond a moderate level should produce smaller-than-expected increases in behavioral influence or no additional enhancement.

### Method

#### Participants

Thirty-two undergraduate students from a local university participated in Experiment 4 (28 female; mean age = 20.94 years, range = 18–26). All participants reported normal or corrected-to-normal vision and provided written informed consent prior to participation. The study was approved by the Institutional Review Board of the authors’ university and conducted in accordance with the Declaration of Helsinki.

Sample size was determined based on the effect sizes observed in Experiment 3 and previous research examining congruity effects in numerical cognition (Matthews and Lewis, 2017). Because the primary theoretical prediction concerned higher-order interactions involving congruity and scaling magnitude, the design emphasized sufficient trial-level observations within participants to support mixed-effects and computational modeling analyses.

#### Design

The experiment employed a 2 (ratio distance: near, far) × 2 (perceptual congruity: congruent, incongruent) × 2 (scaling magnitude: 2-fold, 5-fold) fully within-subjects design. Perceptual congruity referred to whether scaling manipulations supported or conflicted with the relational judgment required by the task. Scaling magnitude parametrically manipulated the strength of the perceptual cue. The experiment used the same eight ratio pairs presented in the previous experiments (Table 1). Combining ratio distance, congruity, scaling magnitude, and spatial counterbalancing produced 32 unique trial types. Each trial type was repeated eight times, yielding a total of 256 trials per participant. Trials were presented in randomized order across two experimental blocks separated by a short break.

#### Stimuli

Stimuli were adapted from Experiment 3 and consisted of pairs of non-symbolic proportions constructed from vertically arranged white and gray circles. The proportional relationships remained identical across conditions, while continuous perceptual magnitude was manipulated bidirectionally.

In congruent trials, perceptual magnitude cues supported the correct proportional judgment. Specifically, the stimulus with the smaller proportion value was enlarged while the stimulus with the larger proportion value was reduced. In incongruent trials, this transformation was reversed: the stimulus with the larger proportion value was enlarged while the smaller proportion value was reduced, creating conflict between perceptual magnitude cues and relational information.

The strength of perceptual manipulation was parametrically varied. In the 2-fold condition, enlarged stimuli were scaled by a factor of 2 and reduced stimuli by a factor of 1/2. In the 5-fold condition, enlarged stimuli were scaled by a factor of 5 and reduced stimuli by a factor of 1/5. For the 2-fold scaling condition, the area range was identical to Experiment 3 (1,400–50,400 pixels). For the 5-fold scaling condition, the manipulation produced a larger range of single-circle areas (560–126,000 pixels). These transformations substantially increased perceptual magnitude differences while preserving the proportional relationship between the two component areas.

#### Procedure

The procedure was identical to that of Experiment 3. Each trial began with a fixation cross presented for 500 ms, followed by a blank interval of 250–300 ms. The stimulus pair was then displayed for up to 1500 ms. Participants were instructed to indicate which stimulus contained the larger proportion of gray circles by pressing the “F” key for the left stimulus or the “J” key for the right stimulus as quickly and accurately as possible. No feedback was provided. The location of the larger proportion and the congruity condition were counterbalanced across trials. Participants were instructed to indicate which stimulus represented the larger proportion value by comparing the relationship between the upper white circle and the lower gray circle within each stimulus.

#### Dependent measures and data analysis

Accuracy was analyzed using GLMMs with a binomial link function, whereas RTs for correct trials were analyzed using LMMs. Trials with RTs shorter than 200 ms or exceeding 3 standard deviations above the participant-specific mean were excluded prior to analysis. The critical analysis focused on whether the influence of perceptual cue strength varied as a function of scaling magnitude. Under a linear integration account, increasing perceptual magnitude should produce corresponding increases in congruity effects. Alternatively, if perceptual integration is constrained, stronger cues may produce smaller-than-expected increases in behavioral influence or altered decision dynamics.

To further characterize the computational mechanisms underlying these effects, trial-level data were additionally analyzed using hierarchical DDM. Competing models were used to evaluate whether perceptual information was better characterized by linear or bounded transformations during evidence accumulation. Specifically, perceptual signals were modeled either as linearly contributing to drift rate or as undergoing a bounded non-linear transformation prior to integration. Model comparison was conducted using WAIC to evaluate which computational architecture better accounted for the joint distribution of choices and response times. Furthermore, these competing computational accounts and hierarchical drift-diffusion modeling analyses were conducted and are reported in a separate Computational Modeling section.

### Results

For the accuracy analysis, trials with no behavioral response were excluded. Of the 8,192 raw trials, 74 trials (0.90%) had no response and were removed, leaving 8,118 trials for the accuracy analysis. For the RT analysis, these no-response trials were first removed, after which trials exceeding each participant’s mean RT by more than 3 SDs were excluded. This procedure removed an additional 45 trials, resulting in 119 exclusions in total (1.45%) and leaving 8,073 RT-valid trials. Because RT analyses were restricted to correct responses, 6,086 trials were ultimately included in the RT model. Condition-specific trial counts and descriptive statistics are reported in Supplementary Table 4.

#### Accuracy

Accuracy was analyzed using a GLMM with a binomial distribution and logit link function. Ratio distance (near vs. far), perceptual–relational congruity (congruent vs. incongruent), bidirectional scaling strength (moderate vs. strong), and all interactions were entered as fixed effects, with random intercepts for participants (Table 8). Moderate and strong scaling corresponded to the 2-fold and 5-fold bidirectional scaling manipulations, respectively.

**TABLE 8:** Generalized linear mixed-effects model results for accuracy in Experiment 4.

Predictor	Estimate (β)	SE	z	*p*
Intercept	1.347	0.084	16.01	<0.001
Ratio distance (Far)	3.393	0.344	9.87	<0.001
Incongruent (vs. Congruent)	−1.826	0.101	−18.00	<0.001
Scaling magnitude (Strong)	−0.172	0.107	−1.60	0.110
Ratio distance × Incongruity	−0.530	0.368	−1.44	0.150
Ratio distance × Strong	−0.584	0.421	−1.39	0.166
Incongruity × Strong	−0.020	0.142	−0.14	0.890
Ratio distance × Incongruity × Strong	0.203	0.455	0.45	0.656

Congruent trials, near-ratio distance, and 2-fold scaling served as reference levels. Positive coefficients indicate higher log-odds of correct responses.

The analysis revealed a significant main effect of ratio distance, β = 3.393, *SE* = −344, *z* = 9.87, *p* < 0.001, with higher accuracy for far than near ratios. There was also a significant effect of congruity, β = −1.826, SE = 0.101, *z* = −18.00, *p* < 0.001, indicating lower accuracy on incongruent than congruent trials under the reference condition. By contrast, the main effect of scaling strength was not significant, β = −0.172, SE = 0.107, *z* = −1.60, *p* = 0.110.

Importantly, neither the congruity × scaling-strength interaction, β = −0.020, SE = 0.142, z = −0.14, *p* = 0.890, nor the three-way interaction among ratio distance, congruity, and scaling strength, β = 0.203, SE = 0.455, z = 0.45, *p* = 0.656, was significant. The ratio distance × congruity interaction, *p* = 0.150, and the ratio distance × scaling-strength interaction, *p* = 0.166, were also not significant. Thus, on the model’s logit scale, the accuracy data did not provide evidence that increasing bidirectional scaling strength systematically altered the congruity effect.

For far ratios, predicted accuracy was 0.991 [0.983, 0.995] for congruent and 0.916 [0.896, 0.932] for incongruent trials under moderate scaling, compared with 0.982 [0.971, 0.988] and 0.860 [0.835, 0.881], respectively, under strong scaling. Congruity differences were again significant under both moderate scaling, estimate = −0.076, 95% CI [−0.094, −0.057], *p* < 0.001, and strong scaling, estimate = −0.122 [−0.146, −0.098], *p* < 0.001 (Figure 5A).

**FIGURE 5:**
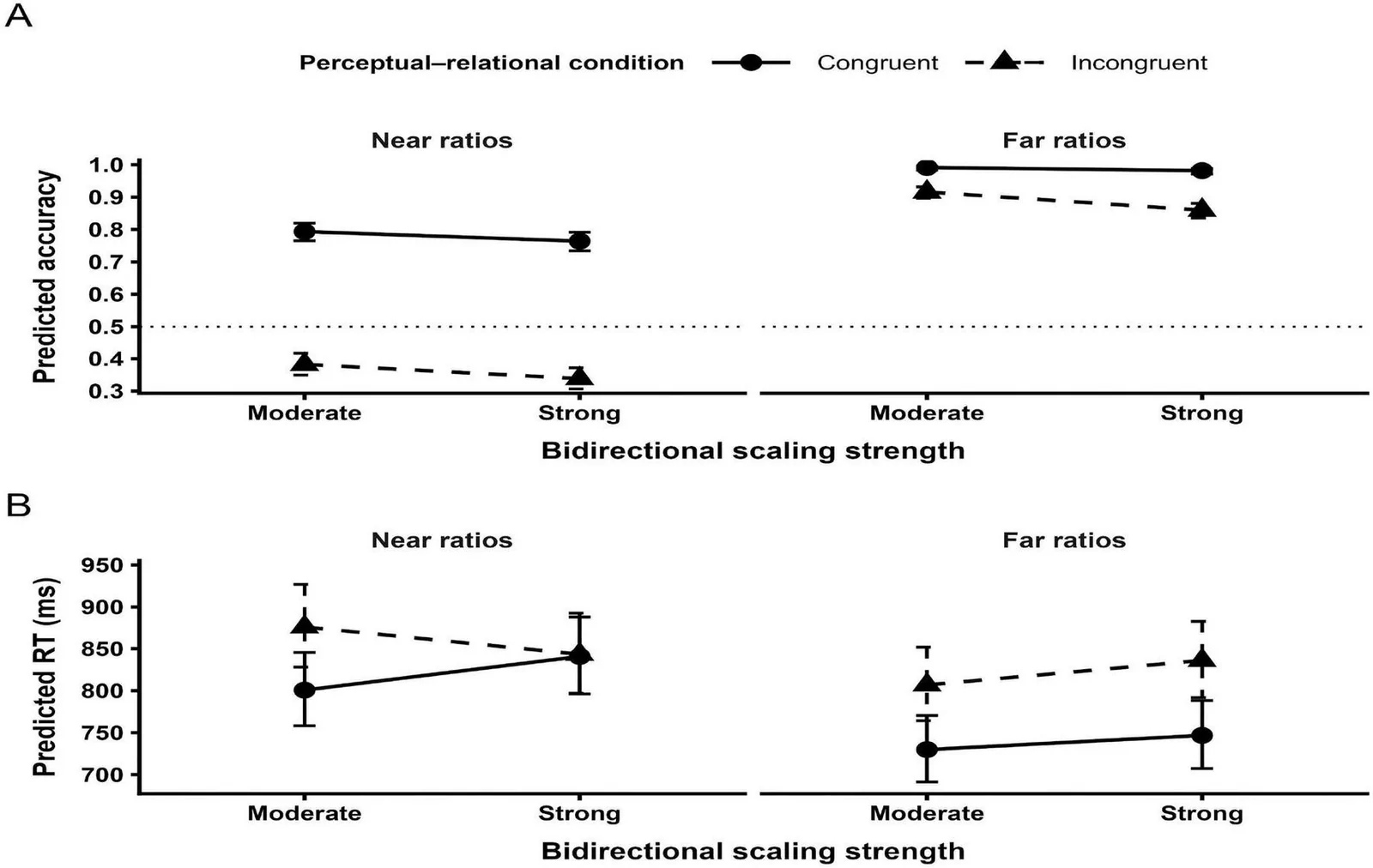
Effects of bidirectional scaling strength and perceptual-relational congruity on non-symbolic proportion judgments in Experiment 4. **(A)** Shows model-estimated accuracy, and **(B)** shows model-estimated reaction times for correct trials. Moderate and strong conditions correspond to 2-fold and 5-fold bidirectional scaling manipulations, respectively. Separate panels show near- and far-ratio conditions. The dotted horizontal line in Panel A indicates chance-level performance (0.50). Error bars represent model-based 95% confidence intervals.

Probability-scale follow-up comparisons showed that the magnitude of the congruity difference did not differ between strong and moderate scaling for near ratios, difference = −0.015, 95% CI [−0.070, 0.041], *p* = 0.608. For far ratios, the probability-scale congruity difference was modestly larger under strong than moderate scaling, difference = −0.046, 95% CI [−0.076, −0.017], *p* = 0.002 (Supplementary Figure 4A). Because this response-scale comparison occurs after the nonlinear inverse-logit transformation, however, it does not alter the absence of significant congruity × scaling or three-way interactions on the model’s logit scale.

Overall, therefore, accuracy showed a strong and reliable effect of perceptual–relational congruity, but increasing bidirectional scaling from moderate to strong did not produce a uniform amplification of this effect.

#### Reaction time

RTs from correct trials were log-transformed and analyzed using a LMM with the same fixed-effects structure and random intercepts for participants (Table 9). As noted above, 6,086 correct trials entered the RT model.

**TABLE 9:** Linear mixed-effects model results for log-transformed response time in Experiment 4.

Predictor	Estimate (β)	SE	df	t	*p*
(Intercept)	6.779	0.030	29.232	226.890	<0.001
Ratio distance (far)	−0.084	0.016	32.001	−5.325	<0.001
incongruity (vs. congruent)	0.087	0.016	33.396	5.592	<0.001
Scaling magnitude (5-fold)	−0.040	0.021	24.941	−1.841	0.077
Ratio distance × incongruent	−0.013	0.018	52.314	−0.739	0.463
Ratio distance × scaling magnitude	0.078	0.022	31.373	3.503	0.001
Incongruent × scaling magnitude	0.090	0.021	34.439	4.368	<0.001
Ratio distance × incongruent × scaling magnitude	−0.107	0.025	60.672	−4.346	<0.001

Congruent trials, near-ratio trials, and moderate bidirectional scaling served as the reference levels. Positive coefficients indicate longer log-transformed RTs.

The RT analysis revealed a significant main effect of ratio distance, β = −0.093, SE = 0.009, *t* = −10.05, *p* < 0.001, indicating faster responses for far than near ratios. Incongruent trials were also slower than congruent trials under the reference condition, β = 0.090, SE = 0.012, *t* = 7.42, *p* < 0.001. In addition, strong scaling was associated with longer RTs than moderate scaling under the reference condition, β = 0.049, SE = 0.010, *t* = 4.96, *p* < 0.001.

More importantly, the congruity × scaling-strength interaction was significant, β = −0.087, SE = 0.018, *t* = −4.95, *p* < 0.001, and was further qualified by a significant three-way interaction among ratio distance, congruity, and scaling strength, β = 0.100, SE = 0.022, *t* = 4.61, *p* < 0.001. The ratio distance × scaling-strength interaction was also significant, β = −0.026, SE = 0.013, *t* = −1.98, *p* = 0.048, whereas the ratio distance × congruity interaction was not, *p* = 0.494. These interactions indicate that the effect of scaling strength on congruity-related RT differences depended on ratio distance.

Follow-up comparisons clarified the three-way interaction. Under moderate scaling, incongruent responses were significantly slower than congruent responses for both near ratios, estimate = 0.090, SE = 0.012, z = 7.42, *p* < 0.001, and far ratios, estimate = 0.100, SE = 0.009, z = 11.35, *p* < 0.001. Model-estimated RTs were 800.7 ms for congruent and 876.1 ms for incongruent near-ratio trials, and 729.8 ms and 806.8 ms, respectively, for far-ratio trials.

Under strong scaling, however, the congruity effect differed sharply across ratio distances. For near ratios, the incongruent–congruent contrast was no longer significant, estimate = 0.003, SE = 0.013, z = 0.24, *p* = 0.807; estimated RTs were virtually identical for congruent (840.8 ms) and incongruent (843.4 ms) trials. In contrast, a robust congruity effect remained for far ratios, estimate = 0.113, SE = 0.009, z = 12.52, *p* < 0.001, with estimated RTs of 746.7 ms for congruent and 836.1 ms for incongruent trials (Figure 5B).

Direct comparisons of congruity-effect magnitude across scaling strengths further confirmed this dissociation. For near ratios, the incongruent–congruent RT difference was significantly reduced under strong relative to moderate scaling, estimate = −0.087, SE = 0.018, z = −4.95, *p* < 0.001. Expressed as percentage slowing, the interference effect decreased from approximately 9.4% under moderate scaling to 0.3% under strong scaling. By contrast, the corresponding change for far ratios was not significant, estimate = 0.013, SE = 0.013, z = 1.01, *p* = 0.315; the estimated interference effect was approximately 10.6% under moderate scaling and 12.0% under strong scaling (Supplementary Figure 4B).

Because the RT analysis was restricted to correct responses, these estimates characterize response speed conditional on successful judgments rather than the full distribution of trial outcomes. This consideration is particularly relevant for the near-ratio incongruent conditions, which contributed fewer correct RT observations than the other conditions.

Taken together, the accuracy and RT results show that increasing bidirectional scaling strength did not produce a uniform increase in perceptual–relational interference. Accuracy remained strongly sensitive to congruity at both scaling strengths, whereas RT revealed a ratio-distance-dependent change: the congruity-related slowing observed under moderate scaling was strongly attenuated under strong scaling for near ratios but remained robust for far ratios. Thus, the behavioral consequences of stronger continuous perceptual magnitude cues were neither uniformly amplified nor expressed identically across choice accuracy and response time. The computational analyses reported below evaluate whether this non-uniform pattern is better captured by linear or bounded integration architectures.

### Discussion

Experiment 4 tested whether stronger continuous perceptual magnitude cues produce progressively larger effects on non-symbolic proportion judgments. Moderate (2-fold) and strong (5-fold) bidirectional scaling were compared under congruent and incongruent conditions. Congruity reliably affected both accuracy and RT, consistent with evidence that continuous visual properties can influence numerical and proportional judgments even when they are not directly relevant to the instructed comparison (Gebuis and Reynvoet, 2012a; Leibovich et al., 2017; Matthews and Lewis, 2017). However, increasing scaling strength did not uniformly amplify these effects.

The accuracy results showed robust congruity effects at both scaling strengths, but neither the congruity × scaling interaction nor the three-way interaction with ratio distance was significant. Thus, stronger scaling did not produce a reliable monotonic increase in categorical choice effects. The RT results revealed a more selective pattern. Under moderate scaling, incongruent trials were slower than congruent trials for both near and far ratios. Under strong scaling, this RT congruity effect was almost eliminated for near ratios but remained robust for far ratios. Because ratio distance indexes relational discriminability, with near ratios imposing greater comparison difficulty than far ratios (Aulet and Lourenco, 2023a; Boyer and Levine, 2012; Matthews et al., 2016), these findings indicate that the influence of cue strength depends on the difficulty of the relational comparison.

This pattern is difficult to reconcile with a simple monotonic account in which stronger perceptual signals continuously increase their behavioral influence. Instead, it is consistent with the possibility that perceptual–relational integration is functionally constrained. Classical cue-integration models predict greater weighting of stronger or more reliable information sources (Ernst and Banks, 2002; Ernst and Bülthoff, 2004), but the present results suggest that increasing perceptual strength does not necessarily translate into proportionally greater influence. Importantly, the data do not demonstrate global saturation: strong scaling attenuated the RT congruity effect for near ratios but not for far ratios. The constraint therefore appears context dependent rather than uniform.

The dissociation between accuracy and RT further suggests that cue-strength effects may be expressed differently across behavioral measures. Accuracy remained strongly sensitive to congruity, whereas RT showed a marked ratio-distance-dependent change. Because accuracy and RT reflect partly distinct components of decision formation (Forstmann et al., 2016; Ratcliff and McKoon, 2008), this dissociation motivates a joint computational analysis rather than relying on either measure alone. Across the four experiments, the results therefore support a context-sensitive perceptual–relational integration account in which the influence of continuous visual information depends on congruity, relational difficulty, and cue strength, rather than increasing indefinitely with perceptual magnitude.

## Computational modeling

### Formalizing perceptual-relational integration

To further characterize the latent decision processes underlying perceptual-relational integration, we implemented hierarchical drift-diffusion models that compared alternative assumptions about how perceptual information contributes to evidence accumulation. The DDM provides a process-level account of decision making by jointly modeling response accuracy and response time as the outcome of noisy evidence accumulation toward decision boundaries (Forstmann et al., 2016; Kang and Ratcliff, 2020; Ratcliff and McKoon, 2008). The computational framework is illustrated in Figure 6.

**FIGURE 6:**
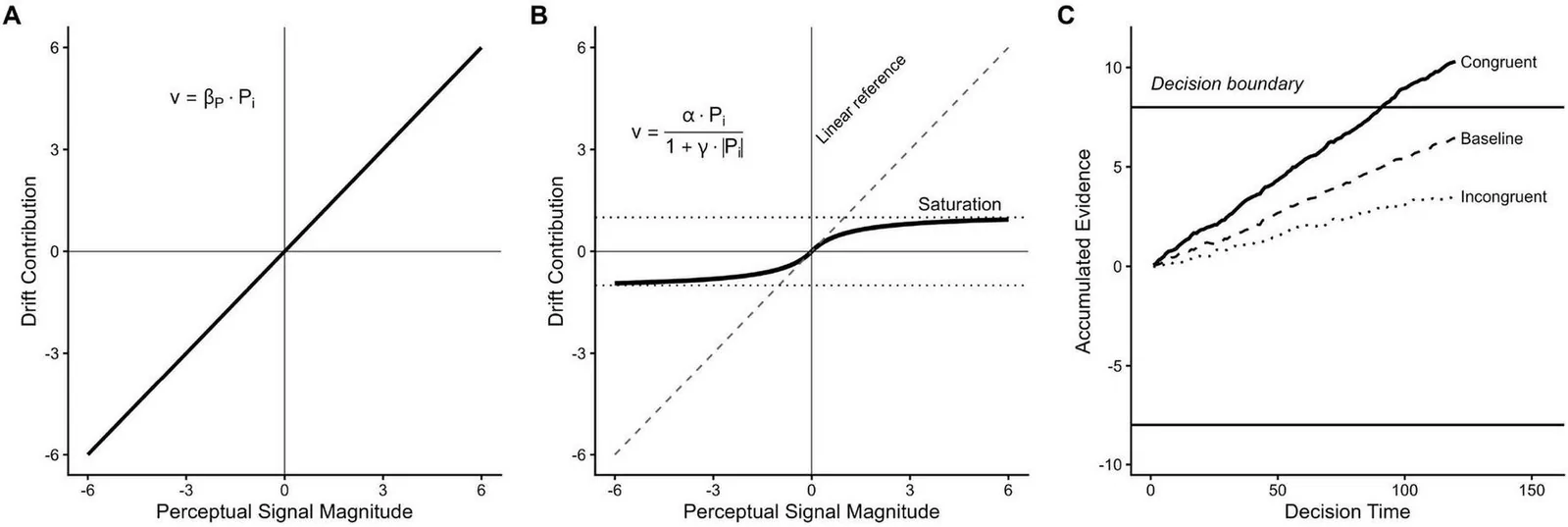
Computational framework of proportion judgments. **(A)** The linear integration model assuming a constant drift rate contribution, **(B)** The proposed bounded integration model where the drift contribution saturates at extreme signal magnitudes according to the function ${\text{v}}_{\text{i}}=\beta_{\text{r}}\,{\text{R}}_{\text{i}}+\alpha \cdot \left(\frac{{\text{P}}_{\text{i}}}{1+\gamma \,\left|{\text{P}}_{\text{i}}\right|}\right)$, **(C)** Schematic illustration of evidence accumulation trajectories of the Diffusion Decision Model (DDM) for congruent, baseline, and incongruent conditions.

Before introducing the process-level implementation, we first formalized the decision computation at an abstract level. We assumed that, on each trial *i*, participants computed a latent decision variable reflecting the combined influence of relational and perceptual information, as shown in Equation 1:


$$D_{i}=w_{R}\,R_{i}+w_{A}\,g\,\left(P_{i}\right)+\varepsilon_{i}$$ (1)

where R_i_ denotes the signed relational signal (e.g., log-transformed ratio difference), P_i_ denotes the signed continuous perceptual magnitude signal (e.g., log-transformed area difference), w_R_ and w_A_ represent the corresponding contributions of relational and perceptual information, and ε_i_ captures stochastic decision noise. The abstract decision variable formulation above provides an intuitive characterization of how relational and perceptual information jointly contribute to choice tendencies.

Additionally, to account for both accuracy and response time, we embedded this decision variable within a DDM framework. Specifically, the trial-wise drift rate *v_i_*, which determines the speed and direction of evidence accumulation, as shown in Equation 2:


$$v_{i}=w_{R}\,R_{i}+w_{A}\,g\,\left(P_{i}\right)$$ (2)

Thus, the DDM serves as a process-level implementation of the same underlying integration computation.

### Linear integration model

As a baseline, we first implemented a standard linear integration model, as shown in Equation 3:


$$v_{i}=\beta_{r}\,R_{i}+\beta_{p}\,P_{i}$$ (3)

The linear model served as a parsimonious benchmark in which relational and perceptual information contributed additively to evidence accumulation, with the contribution of perceptual information increasing proportionally with perceptual signal magnitude. It was intended as a computational benchmark for monotonic integration rather than as a direct implementation of any single normative cue-integration theory (Ernst and Banks, 2002; Gold and Shadlen, 2007). Under this framework, increases in perceptual cue magnitude are expected to produce proportional increases in the contribution of perceptual information to the decision variable.

### Bounded integration model

To examine whether perceptual contributions may be constrained at higher cue magnitudes, we implemented a bounded transformation of perceptual input, as shown in Equation 4:


$$v_{i}=\beta_{r}\,R_{i}+\alpha \cdot \left(\frac{P_{i}}{1+\gamma \,\left|P_{i}\right|}\right)$$ (4)

Where α controls the overall contribution of perceptual information, and γ > 0 determines the degree of saturation. For relatively small values of P_i_, the function approximates linearity (g(P_i_)≈P_i_), whereas larger perceptual differences are progressively compressed, limiting the effective contribution of perceptual information to the accumulation process. This divisive formulation was selected because it preserves local linearity at small signal magnitudes while asymptotically compressing larger perceptual differences, thereby providing a parsimonious implementation of bounded cue influence.

Such divisive normalization-like transformations are consistent with computational principles that are associated with gain-control and normalization processes (Carandini and Heeger, 2011; Louie et al., 2013). Importantly, this formulation provides a parsimonious computational implementation of the bounded integration hypothesis evaluated in the present study. Because Experiment 4 sampled two experimentally defined levels of bidirectional scaling strength, this model comparison should be interpreted as testing a bounded versus linear mapping of perceptual influence rather than as identifying the full form or threshold of a saturation function.

### Parameterization and estimation

To reduce model complexity and ensure stable parameter estimation, only drift rate (*v*) was allowed to vary as a function of experimental variables. Other core DDM parameters were held constant across conditions. Boundary separation (a), starting point (z), and non-decision time (t_0_) were estimated as condition-invariant parameters, with between-subject variability captured through hierarchical random effects. This parameterization follows standard practice when the primary theoretical focus concerns evidence integration rather than strategic speed-accuracy trade-offs (Ratcliff and McKoon, 2008; Vandekerckhove et al., 2011). Hierarchical Bayesian estimation was used to allow partial pooling across participants while preserving individual variability (Wiecki et al., 2013). Additionally, weakly informative priors centered around psychologically plausible parameter ranges were used to stabilize hierarchical estimation.

### Model fitting and comparison

Models were fit to trial-level data from Experiment 4, which uniquely manipulated both perceptual congruity and cue magnitude (2-fold vs. 5-fold scaling). This design provided a direct test of whether perceptual influence scales linearly with cue strength.

For each trial, relational and perceptual signals were operationalized from the trial-level ratio relation and continuous perceptual magnitude difference, respectively, using the transformations specified in the model. Posterior estimation was conducted using hierarchical Bayesian sampling. Convergence diagnostics indicated satisfactory model convergence across chains (all *R* 1.00). Additionally, to evaluate competing accounts, the linear and bounded integration models were compared using expected log predictive density (ELPD) derived from WAIC (Watanabe, 2010). The bounded integration model showed better predictive performance than the linear model (△ELPD = 76.5, SE = 12.3), indicating that a model incorporating a compressive perceptual contribution provided a better account of the observed behavioral distributions among the candidate models considered here. Although this comparison favors the bounded specification over the prespecified linear benchmark, it does not uniquely identify the cognitive mechanism underlying the observed nonlinearity.

Posterior estimates indicated reliable contributions of relational information to evidence accumulation, whereas the contribution of perceptual information was better captured by the bounded transformation. Importantly, the bounded model provided a computational account of the non-uniform behavioral consequences of increasing perceptual cue strength observed in Experiment 4. Increasing bidirectional scaling from moderate (2-fold) to strong (5-fold) did not produce a uniform amplification of congruity effects. Accuracy remained strongly sensitive to congruity across scaling strengths, whereas RT showed a ratio-distance-dependent change, with the congruity-related slowing markedly attenuated under strong scaling for near ratios but preserved for far ratios. Whereas the linear model assumes that larger perceptual signals produce proportionally larger drift contributions, the bounded transformation allows the incremental contribution of increasingly strong perceptual signals to become compressed. This provides a parsimonious account of why stronger perceptual manipulations need not translate into proportionally greater behavioral influence.

As illustrated in Figure 7A, predictive model comparison favored the bounded integration model over the prespecified linear benchmark. Posterior predictive checks further indicated that the bounded model captured key features of the observed behavioral pattern, including changes in RT congruity effects across scaling strengths and ratio-distance conditions (Figure 7B). This predictive advantage supports a compressive account of perceptual contribution but does not uniquely identify the cognitive mechanism responsible for that compression.

**FIGURE 7:**
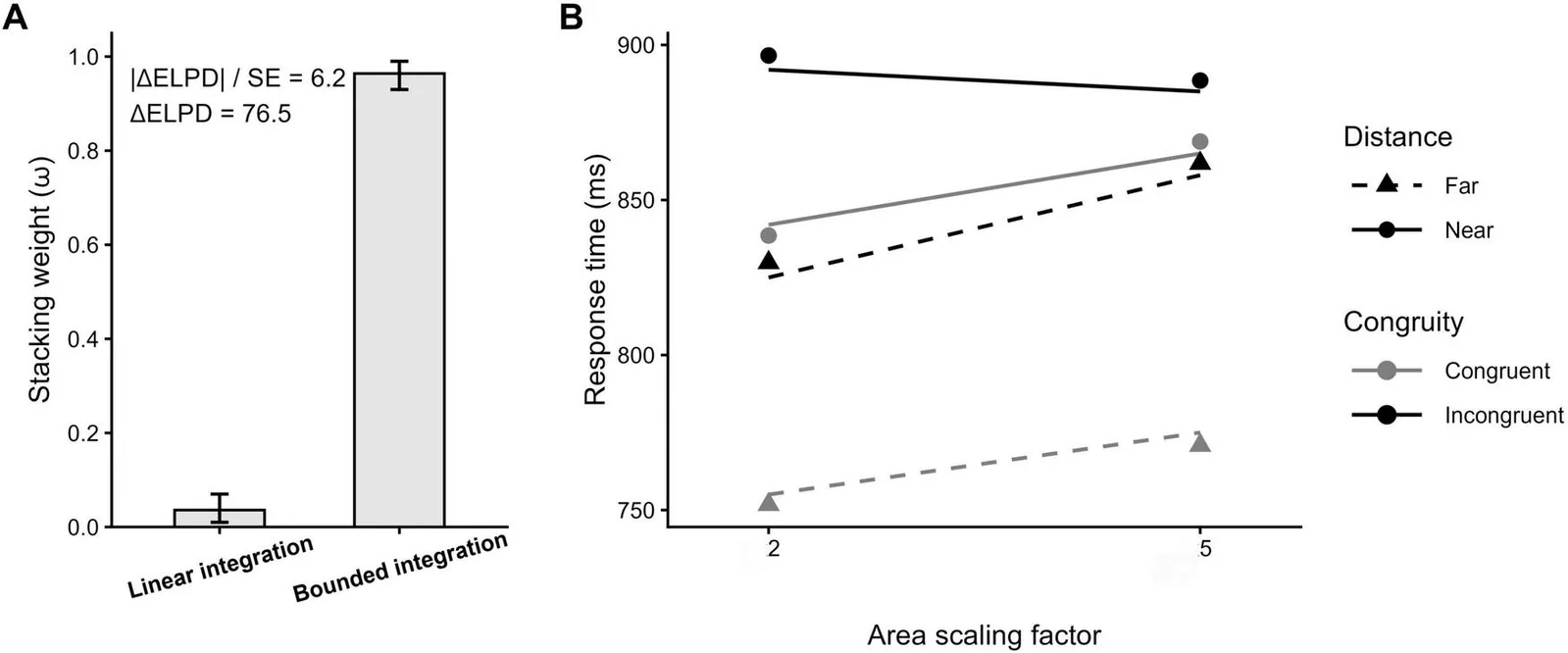
Quantitative evaluation of the bounded integration model. **(A)** Bayesian model selection results favoring the bounded over the linear framework. **(B)** Behavioral validation showing the alignment between observed data and model-predicted RT patterns across experimental conditions.

### Complementary modeling of experiment 3

To examine whether context-sensitive perceptual contributions were also evident outside the cue-strength manipulation of Experiment 4, we applied a related hierarchical DDM framework to Experiment 3. Because Experiment 3 manipulated perceptual–relational congruity at a single bidirectional scaling magnitude, together with a baseline condition, it was not designed to independently estimate the compressive properties evaluated in Experiment 4. Accordingly, this analysis was used as a complementary test of context-sensitive perceptual contributions rather than as an independent test of boundedness.

Consistent with the behavioral results, the modeling indicated that perceptual information contributed differently to evidence accumulation as a function of perceptual–relational congruity. This context dependence accords with the behavioral asymmetry observed in Experiment 3: for near ratios, congruent and incongruent conditions produced bidirectional effects on accuracy, whereas for far ratios the accuracy effect was dominated by an incongruity cost; RT effects likewise differed across ratio-distance and congruity conditions. Thus, Experiment 3 provides complementary evidence that perceptual contributions are not uniformly additive across decision contexts.

Together with the predictive advantage of the bounded model in Experiment 4, these results support a context-sensitive perceptual-relational integration framework. Importantly, the Experiment 3 analysis should not itself be interpreted as evidence for saturation, because cue strength was not parametrically varied in that experiment.

### General discussion

The present study examined how continuous perceptual magnitude information influences non-symbolic proportion judgments by systematically manipulating the direction, congruity, and strength of perceptual signals across four experiments. Across experiments, judgments were reliably affected by perceptual variation even though the underlying proportional relations remained unchanged. At the same time, these effects were neither uniform nor simply proportional to perceptual cue strength. Instead, their behavioral expression depended on ratio distance, perceptual–relational alignment, cue strength, and the measure of performance considered. These findings extend previous work showing that numerical and proportional judgments are sensitive to continuous visual information by demonstrating that such influences are dynamically expressed within a relational decision context (Gebuis et al., 2016; Leibovich et al., 2017; Lourenco and Aulet, 2023; Walsh, 2013).

Taken together, the four experiments point to three broad characteristics of perceptual influence during proportion judgment. First, continuous perceptual information contributes in a context-dependent rather than fixed manner. Second, its behavioral consequences depend on whether perceptual and relational information support or conflict with one another. Third, increasing perceptual signal strength does not produce a uniform or proportional increase in behavioral influence. Together with the computational analyses, these findings are consistent with a context-sensitive and constrained perceptual-relational integration framework, in which relational information remains central to proportion judgment while continuous perceptual information modulates decision formation under context-dependent constraints (see Figure 8).

**FIGURE 8:**
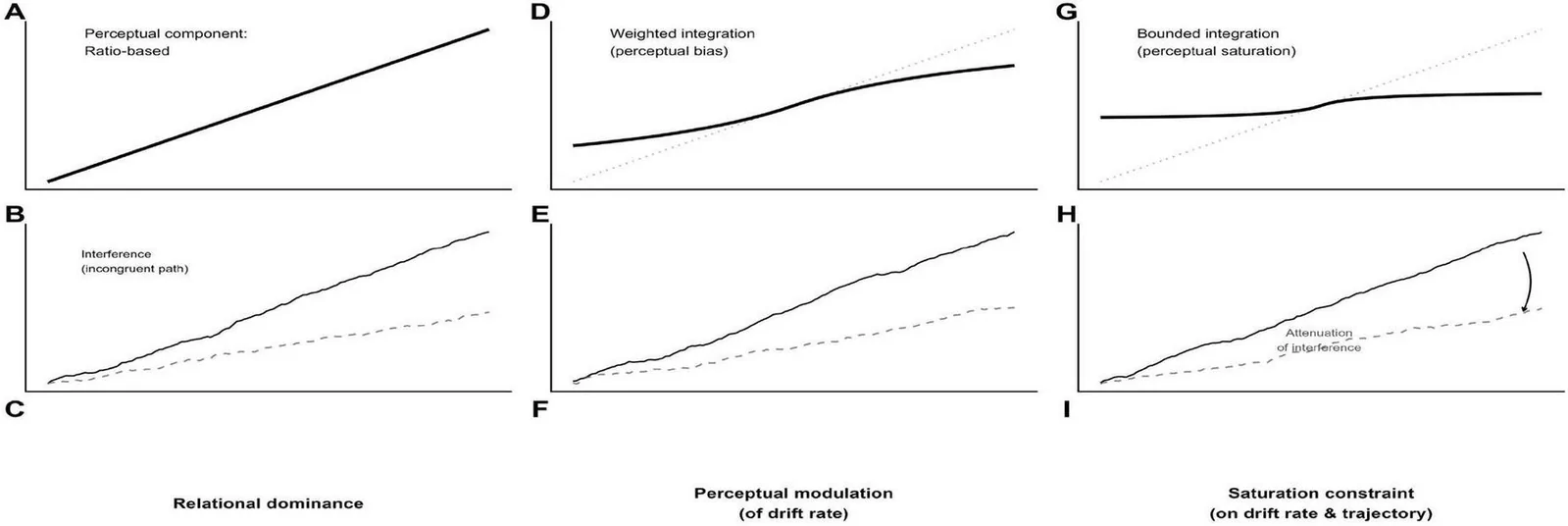
A context-sensitive and constrained perceptual-relational integration framework for non-symbolic proportion judgment. **(A–C)** illustrate a predominantly relational account in which proportion judgments are determined primarily by relational information and perceptual influences are treated as secondary or fixed. **(D–F)** illustrate a linear integration benchmark in which relational and continuous perceptual signals contribute additively to evidence accumulation, such that increasing perceptual signal magnitude produces proportionally greater decision influence. **(G–I)** illustrate the proposed bounded integration account, in which increasingly strong perceptual signals undergo a compressive transformation before contributing to evidence accumulation. Under this framework, stronger perceptual signals need not produce proportionally larger behavioral effects, and their expression may depend on relational discriminability and perceptual–relational alignment. The attenuation of the RT congruity effect under strong scaling for near ratios in Experiment 4 represents one behavioral pattern consistent with such constrained integration, rather than a unique diagnostic signature of saturation.

### Context-dependent influence of continuous perceptual information

Experiments 1 and 2 demonstrated that unilateral changes in continuous perceptual magnitude can alter proportion judgments, but their effects depended on ratio distance and on which competing stimulus was physically transformed. In Experiment 1, enlargement did not produce a uniform directional bias. For near ratios, both enlargement conditions yielded modest improvements in accuracy relative to baseline, whereas for far ratios, enlarging the larger-proportion stimulus impaired performance and enlarging the smaller-proportion stimulus produced little reliable change. Experiment 2 further showed that these effects were not specific to enlargement. Contracting the smaller-proportion stimulus facilitated near-ratio judgments, whereas contracting the larger-proportion stimulus impaired performance, with particularly pronounced disruption under far-ratio conditions.

These findings argue against a simple account in which greater physical extent uniformly increases the perceived proportional value of a stimulus. Instead, continuous perceptual information appears to enter the comparison process in relation to the competing proportional representations. Such context dependence is broadly compatible with cue-integration approaches in which the contribution of auxiliary information depends on the reliability or discriminability of task-relevant evidence (Ernst and Banks, 2002; Gebuis et al., 2016; Ma et al., 2006). However, the present results do not support a simple compensatory rule in which less precise relational representations necessarily produce greater reliance on perceptual information. The direction and magnitude of perceptual effects varied across conditions, suggesting that perceptual weighting depends on the joint structure of relational and perceptual evidence.

Importantly, the directional differences observed in Experiments 1 and 2 should not be interpreted as evidence for a general asymmetric integration mechanism. The task consistently required participants to identify the larger proportion, meaning that transformations applied to the larger- and smaller-proportion stimuli were not equivalent with respect to the response goal. The observed differences are therefore more appropriately characterized as context-dependent asymmetries, whose interpretation remains partly tied to the fixed decision frame.

More broadly, Experiments 1 and 2 indicate that relational and perceptual information should not be treated as mutually exclusive sources of proportion judgment. Robust ratio-distance effects show that participants remained sensitive to proportional structure, whereas systematic effects of perceptual manipulations indicate that relational judgments were not fully insulated from continuous perceptual variation. Perceptual information therefore appears to modulate, rather than replace, relational processing.

### Alignment-and discriminability-dependent integration

Experiment 3 provided a more direct test of how perceptual and relational signals interact by manipulating their congruity bidirectionally. The resulting pattern depended jointly on ratio distance and behavioral measure. For near ratios, accuracy showed bidirectional effects: congruent trials improved performance relative to baseline, whereas incongruent trials impaired it. For far ratios, the incongruity cost remained reliable, whereas the congruent accuracy advantage was absent against an already high baseline level of performance. RT showed a related but non-identical pattern: congruent trials were faster than baseline at both ratio distances, whereas reliable incongruity-related slowing emerged primarily for far ratios.

Thus, congruent and incongruent perceptual information did not produce simple mirror-image effects. Their behavioral consequences depended on relational discriminability and on whether performance was indexed by accuracy or response time. This distinction is theoretically relevant because ratio distance is closely related to the discriminability of proportional relations: near ratios are more difficult to distinguish, whereas far ratios provide more separable relational evidence (Aulet and Lourenco, 2023a; Boyer and Levine, 2012; Matthews et al., 2016). Nevertheless, the present results do not imply that perceptual information is simply weighted more heavily whenever relational information is noisier. Instead, perceptual influence varied according to the joint configuration of ratio distance and perceptual–relational alignment.

The complementary drift-diffusion analysis of Experiment 3 was consistent with this broader interpretation by indicating context-sensitive contributions of perceptual information to evidence accumulation. Perceptual and relational evidence therefore appear to interact within the decision process rather than contributing as two completely independent sources. This interpretation is broadly compatible with evidence-accumulation and conflict-monitoring accounts in which competing information can differentially influence the evolving decision variable depending on its relation to the task-relevant response (Botvinick et al., 2001; Shenhav et al., 2013; Yeung et al., 2004).

Importantly, Experiment 3 manipulated congruity at a single bidirectional scaling magnitude. It therefore provides evidence for alignment-sensitive and context-dependent integration, but should not be interpreted as an independent demonstration of bounded or saturating perceptual influence (Gebuis and Reynvoet, 2012a; Leibovich et al., 2017).

### Cue strength and constrained perceptual influence

Experiment 4 addressed whether stronger continuous perceptual signals produce progressively larger effects on proportion judgment. The results did not support such a simple monotonic amplification account. Accuracy remained strongly sensitive to perceptual–relational congruity under both moderate and strong bidirectional scaling. However, neither the congruity × scaling-strength interaction nor the three-way interaction with ratio distance was significant in the primary GLMM. Thus, increasing cue strength did not reliably amplify congruity effects on categorical choice. Although probability-scale follow-up contrasts indicated a modestly larger congruity difference under strong scaling for far ratios, this effect was not accompanied by a corresponding interaction on the model’s logit scale and therefore should not be interpreted as evidence for a general increase in perceptual influence.

The RT results revealed a more differentiated consequence of cue strength. Under moderate scaling, incongruent trials were slower than congruent trials for both near and far ratios. Under strong scaling, however, the congruity-related RT difference was almost completely attenuated for near ratios while remaining robust for far ratios. Direct comparisons confirmed that increasing cue strength significantly reduced the RT congruity effect for near ratios but did not reliably alter it for far ratios. Stronger perceptual signals therefore did not simply generate greater facilitation or interference. Instead, their temporal consequences depended on relational discriminability.

The divergence between accuracy and RT is particularly informative. Accuracy remained strongly sensitive to congruity across scaling strengths, whereas RT showed a marked ratio-distance-dependent change. Accuracy and response latency can reflect partially distinct aspects of the decision process (Forstmann et al., 2016; Ratcliff and McKoon, 2008), suggesting that changes in cue strength may alter the temporal dynamics of evidence accumulation without producing corresponding changes in final categorical performance. At the same time, this interpretation should remain cautious because conventional RT analyses were restricted to correct responses, particularly affecting difficult incongruent conditions in which fewer observations contributed to the RT estimates.

Overall, the Experiment 4 pattern is more consistent with a constrained than an unrestricted linear account of perceptual contribution. Classical cue-integration principles generally predict greater influence for stronger or more reliable information sources (Ernst and Banks, 2002; Ernst and Bülthoff, 2004). The present findings instead indicate that increasing perceptual signal strength need not translate into a proportional increase in behavioral influence. Importantly, however, the data do not demonstrate global saturation. Strong scaling attenuated the RT congruity effect for near ratios but not for far ratios, while substantial congruity differences in accuracy remained. Any constraint on perceptual contribution therefore appears to be context dependent rather than expressed as a fixed behavioral ceiling across conditions.

The computational analyses provided converging support for this interpretation. Among the prespecified candidate models, the bounded integration model showed better predictive performance than the linear benchmark (ΔELPD = 76.5, SE = 12.3). Whereas the linear model assumes that increasing perceptual signal magnitude produces proportionally larger contributions to evidence accumulation, the bounded model allows the incremental influence of increasingly strong signals to become compressed. This provided a better predictive account of the observed behavioral distributions.

The computational advantage of the bounded model should nevertheless be interpreted at the level warranted by the present design. Experiment 4 included only two experimentally defined levels of bidirectional cue strength and therefore cannot identify the complete functional form or threshold of a saturation process. Moreover, better predictive performance of the bounded model does not uniquely establish a specific normalization mechanism; alternative nonlinear or context-sensitive processes could potentially produce related patterns. The modeling results therefore support bounded or compressive integration as a plausible computational account of constrained perceptual influence rather than as definitive evidence for a unique saturation mechanism.

### A context-sensitive and constrained perceptual–relational integration framework

Taken together, the four experiments support a framework in which non-symbolic proportion judgments emerge from the coordinated contribution of relational and continuous perceptual information. Relational information specifies the task-relevant proportional structure, whereas continuous perceptual information provides auxiliary evidence whose influence depends on relational discriminability, perceptual-relational alignment, and cue strength. Crucially, this perceptual contribution is neither fixed nor assumed to increase indefinitely with signal magnitude.

This framework helps reconcile ratio-based and magnitude-based accounts of non-symbolic proportion processing. Ratio-based approaches correctly emphasize sensitivity to relational structure, as reflected in robust ratio-distance effects across the present experiments (Boyer and Levine, 2012; Jacob et al., 2012; Matthews and Chesney, 2015). At the same time, research on numerical and magnitude processing has shown that judgments involving quantitative information remain sensitive to continuous visual properties such as area, density, spatial extent, and related dimensions (Gebuis et al., 2016; Gebuis and Reynvoet, 2012a; Leibovich et al., 2017). The present findings suggest that these two observations need not be treated as competing explanations.

Specifically, the results do not challenge the existence of relational proportion representations. Rather, they indicate that such representations are not fully perceptually encapsulated during decision making. Relational information appears to determine the task-relevant comparison, whereas continuous perceptual information modulates the accumulation of evidence toward that decision. Depending on the decision context, this auxiliary information may facilitate performance, interfere with judgment, or exert little detectable behavioral effect.

This distinction also clarifies the theoretical status of the proposed bounded integration framework. The central claim is not that perceptual information ceases to contribute once a universal saturation threshold is reached. Instead, the mapping between perceptual magnitude and decision influence may become functionally constrained as cue strength and relational context change. In this sense, boundedness refers to the computational mapping of perceptual evidence rather than to a fixed behavioral ceiling. This formulation accommodates the persistence of strong congruity effects in some conditions alongside attenuation in others.

Accordingly, the framework moves beyond a dichotomy between ratio-based and magnitude-based processing. The more informative question is how relational and perceptual information are weighted and coordinated during decision formation. Across the present experiments, relational structure remained central to performance, but continuous perceptual signals systematically modified the decision process. Their contribution depended on the alignment, discriminability, and strength of the available evidence rather than following a fixed additive rule.

## Limitations and future directions

Several limitations qualify these conclusions. First, the scaling manipulations necessarily changed multiple correlated continuous visual properties. Although cumulative area constituted the primary manipulated dimension, scaling also altered overall physical extent and other characteristics of the stimulus configuration. The present experiments were therefore not designed to isolate a single low-level visual feature. The results are more appropriately interpreted as evidence for the contribution of continuous perceptual magnitude information broadly construed. This interpretation is consistent with proposals that numerical judgments may reflect interactions among multiple correlated perceptual dimensions rather than reliance on an isolated visual cue (Gebuis and Reynvoet, 2012a; Leibovich et al., 2017). Future work should manipulate area, spatial extent, density, and related visual dimensions more selectively to determine their relative contributions.

Second, the task consistently required participants to identify the larger proportion. This fixed response frame may have contributed to some of the directional differences observed when the larger- versus smaller-proportion stimulus was transformed, particularly in Experiments 1 and 2. Consequently, these asymmetries cannot be attributed exclusively to the perceptual manipulation itself. Future studies should counterbalance the decision rule or include smaller-proportion judgments to determine whether similar effects generalize across response goals.

Third, Experiment 4 compared only two levels of bidirectional cue strength. Although this manipulation provided a direct test of whether a substantial increase in perceptual magnitude produced proportional behavioral amplification, it was insufficient to map the full functional relationship between perceptual magnitude and decision influence. A denser parametric manipulation incorporating intermediate and more extreme cue strengths would provide a stronger test of linear, compressive, saturating, and potentially non-monotonic functions.

Fourth, several far-ratio conditions approached high levels of accuracy. In particular, the absence of an additional congruent accuracy benefit under some far-ratio conditions should therefore be interpreted in light of the limited room for further improvement. Such ceiling effects may partially constrain comparisons of facilitative effects across ratio distances.

Fifth, conventional RT analyses were based on correct responses. This is especially relevant for difficult incongruent conditions, in which fewer trials contributed to the RT estimates. Accordingly, the RT results characterize response speed conditional on successful judgments and should be interpreted jointly with accuracy rather than as an independent measure of processing efficiency. Process-level models that jointly characterize choice and response-time distributions provide an important complementary approach for future work (Ratcliff and McKoon, 2008).

Finally, the present computational analyses compared a prespecified linear benchmark with a bounded alternative. Although the bounded model showed superior predictive performance, this comparison does not rule out other nonlinear or context-dependent formulations. Future research combining denser cue-strength manipulations with broader model comparison will be important for identifying the computational form that best characterizes perceptual-relational integration.

## Conclusion

Across four experiments, continuous perceptual magnitude information systematically influenced non-symbolic proportion judgments even though the underlying proportional relations remained unchanged. These effects varied with ratio distance, perceptual–relational alignment, cue strength, and behavioral measure, demonstrating that perceptual contributions are context sensitive rather than fixed.

Critically, stronger perceptual signals did not produce a uniform increase in behavioral influence. Experiment 3 showed that perceptual-relational alignment produced ratio-distance- and measure-dependent behavioral effects, while Experiment 4 showed that increasing cue strength altered those effects in a non-uniform manner. Together with the predictive advantage of the bounded computational model over the prespecified linear benchmark, these findings are consistent with a constrained perceptual-relational integration account.

Relational information therefore remains central to non-symbolic proportion judgment, but it is not fully insulated from continuous perceptual information. Rather than treating ratio-based and magnitude-based explanations as mutually exclusive, the present findings suggest that proportion judgments emerge from their dynamic coordination during decision formation. Continuous perceptual signals modulate relational judgments, but the magnitude and expression of this influence depend on the structure and strength of the available evidence.

### Constraints on generality (COG)

The present findings were obtained from Chinese university students performing a non-symbolic proportion comparison task involving visual perceptual magnitude conditions. Accordingly, the generalizability of the observed bounded integration effects to other populations, age groups, or educational backgrounds remains to be established. In addition, the present experiments manipulated only cumulative area as the continuous perceptual cue. It is therefore unclear whether similar bounded integration dynamics would emerge for other continuous dimensions relevant to numerical cognition, such as density, contour length, or convex hull. More broadly, the current conclusions are limited to non-symbolic visual proportion judgments and may not generalize directly to symbolic proportional reasoning or real-world decision contexts. Finally, the present study tested a restricted range of scaling magnitudes (2-fold and 5-fold); future work will be necessary to characterize the full functional form of perceptual integration across a wider cue-strength continuum.

## Data Availability

The datasets presented in this study can be found in online repositories. The names of the repository/repositories and accession number(s) can be found in the article/Supplementary material.

## Footnotes

1
https://osf.io/nuk7v/overview?view_only=708377b27a6d4301aec61dc9e7fe4ef1

## Supplementary material

Supplementary Figure 1 Changes in predicted accuracy relative to baseline in Experiment 1. Points represent model-estimated changes in accuracy for the larger-proportion-enlarged and smaller-proportion-enlarged conditions relative to baseline, separately for near- and far-ratio trials. Positive values indicate higher predicted accuracy than baseline, whereas negative values indicate lower predicted accuracy. Error bars represent model-based 95% confidence intervals.

Supplementary Figure 2 Changes in predicted accuracy relative to baseline in Experiment 2. Points represent model-estimated changes in accuracy for the smaller-proportion-contracted and larger-proportion-contracted conditions relative to baseline, separately for near- and far-ratio trials. Positive values indicate higher predicted accuracy than baseline, whereas negative values indicate lower predicted accuracy. Error bars represent model-based 95% confidence intervals.

Supplementary Figure 3 Changes in predicted accuracy and reaction time relative to baseline in Experiment 3. Points represent model-estimated changes in accuracy for the congruent and incongruent conditions relative to baseline, separately for near- and far-ratio trials. Positive values indicate higher predicted accuracy than baseline, whereas negative values indicate lower predicted accuracy. Error bars represent model-based 95% confidence intervals.

Supplementary Figure 4 Comparison of interference-effect magnitude across bidirectional scaling strengths in Experiment 4. Panel A shows the accuracy interference effect, calculated as the model-estimated difference between congruent and incongruent conditions and expressed in percentage points. Panel B shows the RT interference effect, calculated from the model-estimated log-RT contrast between incongruent and congruent conditions and converted to percentage slowing, 100×[exp(β)-1]. Positive values indicate greater interference from perceptual–relational incongruity. Moderate and strong conditions correspond to 2-fold and 5-fold bidirectional scaling manipulations, respectively. Error bars represent model-based 95% confidence intervals. Brackets indicate comparisons of interference-effect magnitude between moderate and strong scaling conditions within each ratio-distance condition; associated *p* values are shown above the brackets. RT analyses were based on correct trials only.
