# Supplementary material for: When more is not more: evidence for bounded perceptual-relational integration in non-symbolic proportion judgments
Source: Front Psychol. 2026 Sep 30;17:1904042. doi: 10.3389/fpsyg.2026.1904042 (PMC13661585; doi:10.3389/fpsyg.2026.1904042)
Supplement: Supplementary file 1 [file Table_1.docx]

Supplementary Table S1. Condition-level descriptive statistics for Experiment 1

| Distance | Area manipulation | Mean ACC (SD) | Mean RT (SD), ms | Valid correct RT n |
| --- | --- | --- | --- | --- |
| Near | Baseline | 57.54% (49.45%) | 905.9 (228.0) | 642 |
| Near | Smaller enlarged | 61.86% (48.59%) | 913.6 (229.5) | 694 |
| Near | Larger enlarged | 62.36% (48.47%) | 922.4 (236.7) | 693 |
| Far | Baseline | 97.01% (17.03%) | 797.0 (195.3) | 1108 |
| Far | Smaller enlarged | 97.45% (15.77%) | 801.0 (200.0) | 1111 |
| Far | Larger enlarged | 94.09% (23.60%) | 803.0 (198.8) | 1071 |

**Note.** ACC = accuracy; RT = reaction time. Accuracy values represent observed trial-level proportions of correct responses. RT values are reported in milliseconds and are based on correct trials only after RT exclusion criteria were applied. Values in parentheses are standard deviations. Valid correct RT n indicates the number of correct-response trials retained for the RT analysis in each condition.
