# Supplementary material for: When more is not more: evidence for bounded perceptual-relational integration in non-symbolic proportion judgments
Source: Front Psychol. 2026 Sep 30;17:1904042. doi: 10.3389/fpsyg.2026.1904042 (PMC13661585; doi:10.3389/fpsyg.2026.1904042)
Supplement: Supplementary file 2 [file Table_2.docx]

Supplementary Table S2. Condition-level descriptive statistics for Experiment 2

| Distance | Area manipulation | Mean ACC (SD) | Mean RT (SD)，ms | Valid correct RT *n* |
| --- | --- | --- | --- | --- |
| Near | Baseline | 57.26% (49.49%) | 947.8 (237.7) | 601 |
| Near | Larger contracted | 49.58% (50.02%) | 990.4 (212.4) | 521 |
| Near | Smaller contracted | 64.05% (48.01%) | 917.3 (263.3) | 666 |
| Far | Baseline | 97.49% (15.66%) | 806.0 (205.5) | 1049 |
| Far | Larger contracted | 47.74% (49.97%) | 866.9 (194.7) | 517 |
| Far | Smaller contracted | 95.81% (20.05%) | 776.8 (207.9) | 1033 |

***Note.*** ACC = accuracy; RT = reaction time. Accuracy values represent observed trial-level proportions of correct responses. RT values are reported in milliseconds and are based on correct-response trials only after application of the RT exclusion criteria. Values in parentheses are standard deviations. Valid correct RT n indicates the number of correct-response trials retained for the RT analysis in each condition.
