# Supplementary material for: When more is not more: evidence for bounded perceptual-relational integration in non-symbolic proportion judgments
Source: Front Psychol. 2026 Sep 30;17:1904042. doi: 10.3389/fpsyg.2026.1904042 (PMC13661585; doi:10.3389/fpsyg.2026.1904042)
Supplement: Supplementary file 3 [file Table_3.docx]

Supplementary Table S3**.** Condition-level descriptive statistics for Experiment 3

| Distance | Congruity | Mean ACC (SD) | Mean RT (SD),ms | Valid correct RT *n* |
| --- | --- | --- | --- | --- |
| Near | Baseline | 59.41% (49.13%) | 956.4 (233.7) | 662 |
| Near | Congruent | 74.45% (43.64%) | 890.4 (232.9) | 838 |
| Near | Incongruent | 46.64% (49.91%) | 938.4 (239.3) | 516 |
| Far | baseline | 94.50% (22.81%) | 824.7 (203.3) | 1067 |
| Far | Congruent | 94.74% (22.34%) | 796.1 (197.7) | 1086 |
| Far | Incongruent | 87.39% (33.21%) | 857.4 (205.0) | 985 |

***Note.*** ACC = accuracy; RT = reaction time. Accuracy values represent observed trial-level proportions of correct responses. RT values are reported in milliseconds and are based on correct-response trials only. Trials with no response, RTs shorter than 200 ms, or RTs exceeding 3 SDs above the participant-specific mean were excluded from the RT analysis. Values in parentheses are standard deviations. Valid correct RT n indicates the number of correct-response trials retained for the RT analysis in each condition.
