# Supplementary material for: When more is not more: evidence for bounded perceptual-relational integration in non-symbolic proportion judgments
Source: Front Psychol. 2026 Sep 30;17:1904042. doi: 10.3389/fpsyg.2026.1904042 (PMC13661585; doi:10.3389/fpsyg.2026.1904042)
Supplement: Supplementary file 4 [file Table_4.docx]

Supplementary Table S4. Condition-level descriptive statistics for Experiment 4

| Distance | Congruity | Bidirectional scaling strength | Mean ACC (SD) | Mean RT (SD), ms | Valid correct RT *n* |
| --- | --- | --- | --- | --- | --- |
| Near | Congruent | 2-fold | 79.15% (40.64%) | 832.4 (227.1) | 795 |
| Near | Congruent | 5-fold | 76.20% (42.61%) | 868.5 (225.0) | 760 |
| Near | Incongruent | 2-fold | 38.33% (48.64%) | 906.2 (221.1) | 383 |
| Near | Incongruent | 5-fold | 33.96% (47.38%) | 873.5 (224.7) | 340 |
| Far | Congruent | 2-fold | 99.11% (9.38%) | 748.4 (174.1) | 1011 |
| Far | Congruent | 5-fold | 98.14% (13.53%) | 768.6 (187.6) | 1003 |
| Far | Incongruent | 2-fold | 91.45% (27.97%) | 832.6 (197.9) | 929 |
| Far | Incongruent | 5-fold | 85.78% (34.94%) | 865.8 (212.4) | 865 |

***Note.*** ACC = accuracy; RT = reaction time. Accuracy values represent observed trial-level proportions of correct responses after exclusion of no-response trials. RT values are reported in milliseconds and are based on correct-response trials only after application of the prespecified RT exclusion criteria. Values in parentheses are standard deviations. Valid correct RT n indicates the number of correct-response trials retained for the RT analysis in each condition. Moderate and strong conditions correspond to 2-fold and 5-fold bidirectional scaling manipulations, respectively.
